# Electrical stimulation alleviates depressive-like behaviors of rats: investigation of brain targets and potential mechanisms

**DOI:** 10.1038/tp.2015.24

**Published:** 2015-03-31

**Authors:** L W Lim, J Prickaerts, G Huguet, E Kadar, H Hartung, T Sharp, Y Temel

**Affiliations:** 1 Department of Biological Sciences, Sunway University, Selangor, Malaysia; 2 Department of Neuroscience, Maastricht University, Maastricht, The Netherlands; 3 Department of Pharmacology, Oxford University, Oxford, UK; 4 Department of Neurosurgery, Maastricht University, Maastricht, The Netherlands; 5 School of Biological Sciences, Nanyang Technological University, Singapore, Singapore; 6 Department of Biology, Girona University, Girona, Spain

## Abstract

Deep brain stimulation (DBS) is a promising therapy for patients with refractory depression. However, key questions remain with regard to which brain target(s) should be used for stimulation, and which mechanisms underlie the therapeutic effects. Here, we investigated the effect of DBS, with low- and high-frequency stimulation (LFS, HFS), in different brain regions (ventromedial prefrontal cortex, vmPFC; cingulate cortex, Cg; nucleus accumbens (NAc) core or shell; lateral habenula, LHb; and ventral tegmental area) on a variety of depressive-like behaviors using rat models. In the naive animal study, we found that HFS of the Cg, vmPFC, NAc core and LHb reduced anxiety levels and increased motivation for food. In the chronic unpredictable stress model, there was a robust depressive-like behavioral phenotype. Moreover, vmPFC HFS, in a comparison of all stimulated targets, produced the most profound antidepressant effects with enhanced hedonia, reduced anxiety and decreased forced-swim immobility. In the following set of electrophysiological and histochemical experiments designed to unravel some of the underlying mechanisms, we found that vmPFC HFS evoked a specific modulation of the serotonergic neurons in the dorsal raphe nucleus (DRN), which have long been linked to mood. Finally, using a neuronal mapping approach by means of c-Fos expression, we found that vmPFC HFS modulated a brain circuit linked to the DRN and known to be involved in affect. In conclusion, HFS of the vmPFC produced the most potent antidepressant effects in naive rats and rats subjected to stress by mechanisms also including the DRN.

## Introduction

Major depression is one of the most common of all psychiatric disorders, with a life-time prevalence of 16.2% for the United States^1^ and 14% for Europe.^2^ It is ranked as a leading cause of societal burden among all diseases. Depression can be treated by antidepressant drugs and/or psychotherapy. In cases with a severe disease course, electroconvulsive therapy is usually applied. Nevertheless, ~60% of subjects remain clinically refractory,^3^ and for these patients, deep brain stimulation (DBS) has been proposed as an effective/potential treatment.^4, 5^

Recent clinical studies have demonstrated that DBS of the subgenual anterior cingulate cortex induced striking and sustained remission of depressive symptoms.^6, 7, 8^ Similarly, DBS of the nucleus accumbens (NAc), a key region in the reward circuitry, produced substantial therapeutic effects in patients with major depression.^9, 10^ These effects were also found with DBS of homolog regions in animal models, namely the ventromedial prefrontal cortex (vmPFC) and NAc.^11, 12, 13, 14, 15^ Besides these two main targets, other brain areas have also been explored as candidates for DBS in clinical case studies including the ventral capsule/ventral striatum^16^ and the lateral habenular nucleus (LHb).^17^ In animal models, these areas include the LHb,^18^ and the ventral tegmental area (VTA).^19^ All of these regions have a critical role in the regulation of negative emotions and are interconnected with a wide range of networks, forming a neurocircuitry for affective disorders.^20, 21^

However, key questions still remain in DBS: (i) Which brain area results in the most optimal behavioral outcome? (ii) Which stimulation parameters are the most effective? (iii) Which mechanisms underlie the therapeutic effects? Here, we addressed these three research questions. We performed DBS with low- or high-frequency stimulation (LFS or HFS) of six different brain areas (vmPFC; cingulate cortex, Cg; NAc core or shell; LHb; and VTA), which have previously been identified in naive animal experiments. For further validation of these findings, we tested the stimulation effects that were obtained from naive animal studies using the chronic unpredictable stress (CUS) animal model of depression. The behavioral effects on anxiety response, food motivation, hedonia and forced-swim immobility for antidepressant activity were assessed. We aimed to cover the various depressive-like symptoms, mimicking clinical depression as far as possible.^22^ In addition to this, the effects of different stimulation parameters were assessed. To investigate possible mechanisms involved, we focused on the midbrain 5-HT (5-hydroxytryptamine, serotonin) system, which has been linked to mood.^23, 24^ We therefore carried out an in-depth examination of the 5-HT neuronal firing and morphology in the dorsal raphe nucleus (DRN) in response to DBS, using both electrophysiological and histochemical approaches. This choice was also supported by previous finding showing a profound effect of DBS on the release of 5-HT in the forebrain.^14^

## Materials and methods

### Design of the study

We conducted two studies to investigate the above-mentioned research questions. In study 1, behavioral experiments were performed on naive animals and in the CUS rat model. A battery of behavioral tests was used to evaluate the effects of DBS with either LFS or HFS. In study 2, since recent studies have shown that vmPFC HFS is the most effective target for antidepressant activity with increased hippocampal 5-HT levels,^14^ we continued with vmPFC HFS, and performed electrophysiological and histochemical studies on the DRN 5-HT neurons to further understand the underlying mechanisms behind antidepressant effects of DBS.

### Study 1

#### Subjects

We used male rats (300–400 g; Sprague Dawley, *n*=212, Charles River, Sulzfeld, Germany), which were housed individually in standard cages on sawdust bedding in a temperature (~20–22 °C) and humidity (60–70%) -controlled environment, using 12/12- h reversed dark/light cycle (lights off at 0800 h). Food and water were available *ad libitum*. All the experiments were carried out in accordance with the Animal Experiments and Ethics Committee of Maastricht University.

Two sets of behavioral experiments were conducted. In the first set of experiments (animals, *n*=124), we stimulated the Cg (LFS, *n*=8; HFS, *n*=8), vmPFC (LFS, *n*=10; HFS, *n*=10), NAc core (LFS, *n*=8; HFS, *n*=8), NAc shell (LFS, *n*=8; HFS, *n*=8), LHb (LFS, *n*=8; HFS, *n*=8), and VTA (LFS, *n*=8; HFS, *n*=8) in naive animals with either LFS or HFS. Sham animals (*n*=24) were implanted in the Cg, vmPFC, NAc core, NAc shell, LHb and VTA (each group, *n*=4), but not stimulated. The behavioral battery included the home-cage emergence test and the open-field test to assess changes in anxiety levels and gross mobility, respectively. The food intake test was applied to measure motivation for food. The sucrose intake test was used to evaluate changes in hedonia, and finally the forced-swim test was used to assess antidepressant-like activity. In the second set of experiments, the most effective DBS targets were tested in the CUS rat model, again using the same behavioral battery. The behavioral sequence of testing for the two sets of experiments was as follows: home-cage emergence test, open-field test, food intake test, sucrose intake test and finally the forced-swim test.

#### Electrode implantation and stimulation procedures

Surgery was performed as previously described.^25, 26^ Detailed information about the surgical procedures can be found in the Supplementary Methods. In brief, a bilateral stimulating electrode was implanted in the Cg (AP: +1.70 mm; L: ±0.60 mm; V: −2.60 mm), vmPFC (AP: +2.70 mm; L: ±0.60 mm; V: −4.60 mm), NAc core (AP: +2.20 mm; L: ±1.50 mm; V: −6.80 mm), NAc shell (AP: +1.70 mm; L: ±0.60 mm; V: −7.20 mm), LHb (AP: −3.80 mm; L: ±0.60 mm; V: −5.00 mm) and VTA (AP: −6.04 mm; L: ±0.60 mm; V: −8.40 mm), according to the rat brain atlas of Paxinos and Watson.^27^ After implantation, animals had a 2-week recovery period.

For DBS, we used a stimulation amplitude of 100 μA on the basis of previous research,^28^ of either LFS (10 Hz) or HFS (100 Hz). The pulse width was set at 100 μs, again on the basis of the results of previous experiments.^29^ A World Precision Instruments digital stimulator (DS8000, WPI, Berlin, Germany) and a stimulus isolator (DLS100, WPI), were used to deliver the stimuli.

#### Behavioral battery

During the behavioral tests, animals received either stimulation (LFS or HFS) or sham stimulation (cables connected but stimulator turned off). In all the experiments, animals were stimulated for ~15 min before each behavioral task and this continued for the entire duration of testing.

Home-cage emergence test: In this test, the home-cage was placed on a platform and the lid of the home-cage was removed. A grid was placed over the edge of the home-cage to make it easier for the animal to leave the home-cage. The experimenters measured the time it took for the rat to climb out of its cage onto the grid. If the rat did not escape from its home-cage within 10 min, the session was ended, and the rat was given a score of 600 s.^30^

Open-field test: The open-field behavior was conducted in an enclosed square, clear, Plexiglas box (100 × 100 × 40 cm), with an open top and a dark floor.^31^ The behavior of each rat was recorded using an automated system consisting of a camera connected to a computer with the EthoVision tracking software (EthoVision, Noldus, The Netherlands). Before testing, the animals were stimulated for 15 min and subsequently tested for 10 min in the open-field arena. A trial was stopped automatically after 10 min and the rat was immediately placed back into its home-cage.

Food intake test: Before testing, the animals were deprived of food and water for 24 h, and stimulated for 15 min before, and then continuously during the entire procedure. The animals were placed in their individual home-cage with access to a limited amount of food in a Petri-dish. After 2 h of testing, the total food intake (g) was calculated from the amount of food consumed.^32^

Sucrose intake test: One day before the test, the animals were habituated to drink 1% sucrose solution by exposing them to sucrose instead of water for 1 h. After a period of 14 h of food and water deprivation, which started at the beginning of the dark phase, animals were offered sucrose for 1 h. The sucrose intake was calculated from the total amount of sucrose solution consumed divided by the weight of the animal (g per kg).^33^

Forced-swim test: This test was carried out using a transparent Perspex cylinder (50 × 20 cm). The cylinder was filled with tap water (25±1 °C) to a depth of 30 cm.^29, 34^ Testing was carried out over two consecutive days. In a pretest session, each rat was placed in the water for 15 min. The following day, the rats were tested in the cylinder containing water for 10 min. Recordings of behavior were taken by a digital camera. The duration of the following behaviors was timed by observers that were masked to treatments: ‘immobility' (no movements or small and infrequent movements performed solely to maintain the nose above the water), ‘swimming' (active swimming with the forepaws) and ‘climbing' (scratching of cylinder walls using both forepaws and hind paws).

#### The chronic unpredictable stress model

The CUS group (HFS vmPFC, *n*=16; HFS NAc core, *n*=16; HFS NAc shell, *n*=16; HFS LHb, *n*=16; sham, *n*=16) was exposed to 3 weeks of chronic unpredictable stressors, whereas operated controls (*n*=8) were left undisturbed. The CUS sham animals received similarly to the naive animals, electrode implantations in the vmPFC, NAc core, NAc shell and LHb (each group, *n*=4), without stimulation. The CUS and the control groups (*n*=88) were placed in separate rooms. The stress procedure was performed according to previous descriptions with some minor modifications.^35, 36^ Briefly, the protocol consisted of intermittent illumination every 2 h, housing in mouse cages, stroboscopic light (2.5 Hz), soiled-cage with 300 ml cold water, paired-housing in dirty cages (with excreta of another rat), food and water deprivation and a condition with no stressors. During paired-housing, rats were grouped in pairs with different partners—alternately a resident or an intruder. All the stressors lasted for 10–14 h. To maintain a low level of predictability, times and order of the CUS were not fixed, but one always took place in the morning and the other in the evening. After 3 weeks of exposure to stress, the stressed and unchallenged control groups were subjected to behavioral testing. Stress was continued during the entire period of testing, but always applied after daily behavioral testing.

### Study 2

#### Subjects

For electrophysiology, we used male rats (270–320 g; Sprague–Dawley, *n*=12, Harlan Olac, Bicester, UK), which were housed in conditions similar to those described in Study 1. All experiments were carried out with approval from the UK Home Office Animals (Scientific Procedures) Act 1986.

For histochemistry, we used the brains of subjects derived from Study 1 of the CUS experiment to compare differences between vmPFC HFS and sham animals.

#### Electrophysiology

Extracellular single-unit recordings of cells in the DRN were performed, as previously described.^37^ Detailed information about the electrophysiology methods used can be found in the Supplementary Methods. In brief, after 5 min of baseline recording of individual DRN neurons, DBS with the most effective parameters (frequency: 100 Hz; amplitude: 100 μA; and pulse width: 100 μs) was performed for 5 min and recordings continued for a further 5 min after the cessation of the stimulus. After recording, the same neuron was then subjected to juxtacellular labeling for postmortem histochemical analysis.

#### Histochemistry

Before being killed, all animals received 1 h HFS followed by 1 h interval in their home-cage. Subsequently, the rats were anesthetized with Nembutal (75 mg kg^−1^) and perfused transcardially with 4% paraformaldehyde fixative solution. The brains were cryoprotected by overnight 15% sucrose treatment and frozen with CO_2_. The brains were cut serially (10 series) on a cryostat (MICROM, Walldorf, Germany) into 30 μm coronal sections and stored at −80 °C until stainings were performed. A standard hematoxylin–eosin (Merck, Darmstadt, Germany) staining was performed to examine the localization of the electrode tips and screen for histological damage. We performed immunohistochemical stainings for c-Fos and 5-HT on the basis of previously established methods.^38, 39^ For the staining methods, microscopic analysis and quantification, please refer to the Supplementary Methods.

#### Statistical analysis

All the data are presented as mean±s.e.m. and statistical analyses were performed with IBM SPSS Statistics 20 (Armonk, New York, USA). Normality and homogeneity of variance was performed using the Kolmogorov–Smirnov test. The data of the behavioral studies were analyzed using one-way analysis of variance with Bonferroni *post hoc* tests for multiple comparisons. Comparisons between CUS-sham and non-CUS control were performed by two-tailed Student's *t*-tests. Electrophysiological data were analyzed by one-way analysis of variance with repeated measures. For the data of the histochemical study, we used either an independent sample *t*-test or a nonparametric Mann–Whitney *U*-test, as appropriate. All *P*-values <0.05 were considered significant.

## Results

### Electrode localization

**Figure 1:**
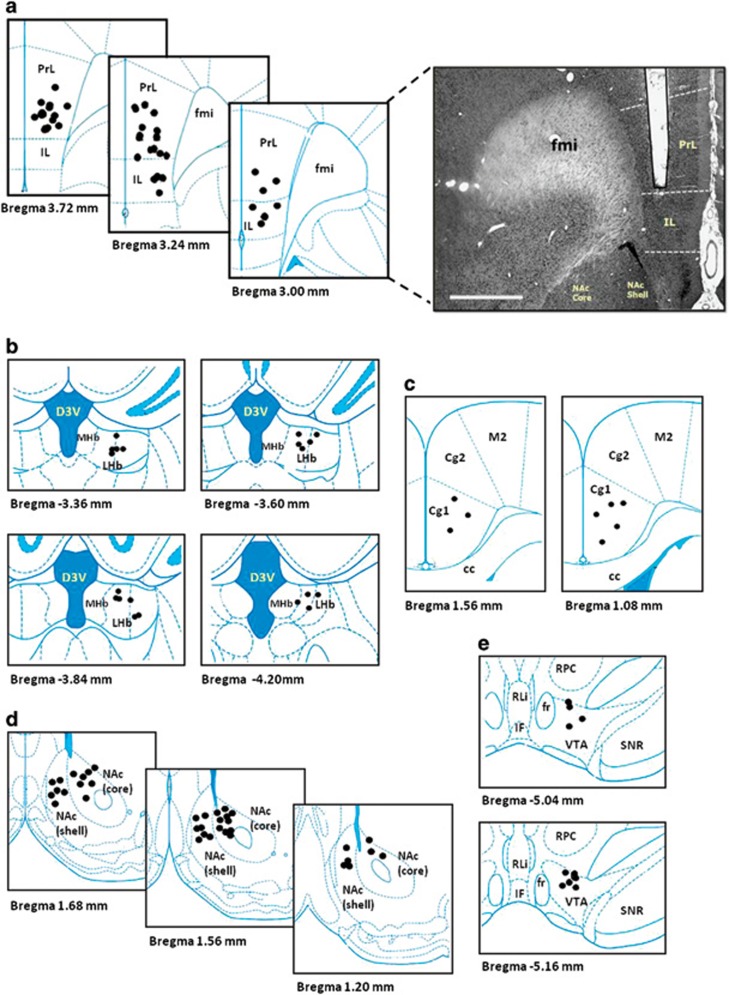
Schematic illustration of the anatomical placement of stimulating electrode in the vmPFC (**a**), LHb (**b**), Cg (**c**), NAc (**d**) and the VTA (**e**). Photomicrograph of a 30-μm-thick coronal section from the brain of a rat showing the histological verification of the electrode location in the vmPFC (scale bar, 1 mm). The symbol (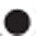) indicates the tips of all electrode localization. cc, corpus callosum; Cg1 and Cg2, cingulate cortex 1 and cingulate cortex 2; D3V, dorsal third ventricle; fmi, forceps minor of corpus callosum; fr, fasciculus retroflexus; IF, interfascicular nucleus; IL, infralimbic; LHb, lateral habenular nucleus; MHb, medial habenular nucleus; M2, secondary motor cortex; NAc core, nucleus accumbens core; NAc shell, nucleus accumbens shell; PrL, prelimbic; RLi, rostral linear nucleus of the raphe; RPC, parvicellular part of the red nucleus; SNR, reticular part of the substantia nigra; vmPFC, ventromedial prefrontal cortex; VTA, ventral tegmental area.

Electrodes were traced in the predefined brain targets in ~92% of the cases. The animals with misplacement or detachment of the electrodes (8% of the cases) in the naive (NAc core HFS, *n*=2; NAc shell HFS, *n*=1; LHb LFS, *n*=2; LFS VTA, *n*=1; sham, *n*=5) and CUS (NAc core HFS, *n*=1; NAc shell HFS, *n*=3; sham, *n*=2) animal experiments were discarded from data analysis. For representative figures and overall electrode positions, see Figure 1. No histological damage was observed except for the electrode trajectory.

Overall, the final number of rats per group was as follows for naive (total, *n*=113; Cg: LFS, *n*=8; HFS, *n*=8; vmPFC: LFS, *n*=10; HFS, *n*=10; NAc core: LFS, *n*=8; HFS, *n*=6; NAc shell: LFS, *n*=8; HFS, *n*=7; LHb: LFS, *n*=6; HFS, *n*=8; VTA: LFS, *n*=7; HFS, *n*=8; and sham, *n*=19) and CUS (total, *n*=82; HFS vmPFC, *n*=16; HFS NAc core, *n*=15; HFS NAc shell, *n*=13; HFS LHb, *n*=16; Sham, *n*=14; and control, *n*=8) animal experiments.

### Study 1

#### Behavioral effects of LFS and HFS in naive animals

**Figure 2:**
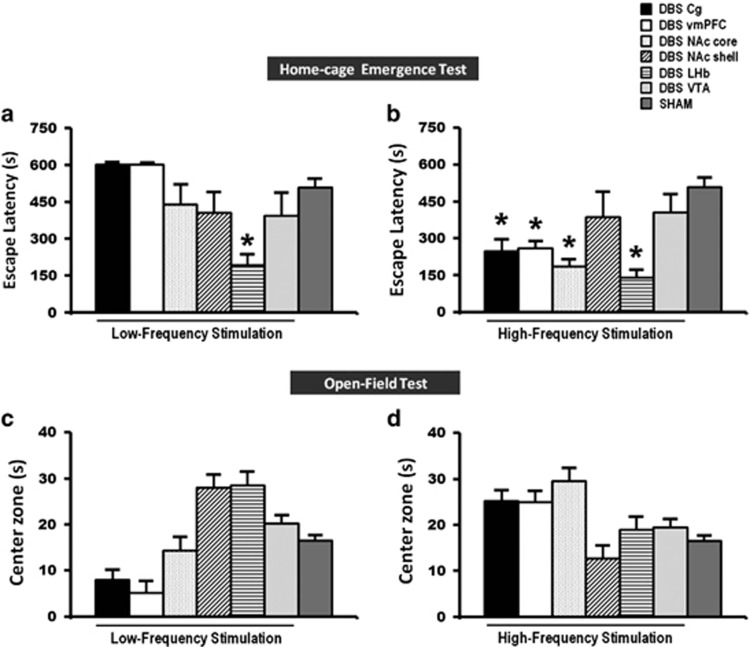
A set of bar graphs showing the measures of anxiety-like behavior by home-cage emergence (**a** and **b**) and open-field (**c** and **d**) tests in naive animal experiments. Before testing, animals were stimulated for 15 min and continuously for another 5 min during both the anxiety tasks. LFS of the LHb, and HFS of the Cg, vmPFC, NAc core and LHb reduced the escape latency from the home-cage emergence test, indicating anxiolytic behavior. No significant difference was found in the open-field test of both LFS and HFS groups. Data represent means±s.e.m. Significant difference from the sham animals, ^*^P<0.05. Cg, cingulate cortex; HFS, high-frequency stimulation; LFS, low-frequency stimulation; LHb, lateral habenular nucleus; NAc, nucleus accumbens; vmPFC, ventromedial prefrontal cortex.

**Figure 3:**
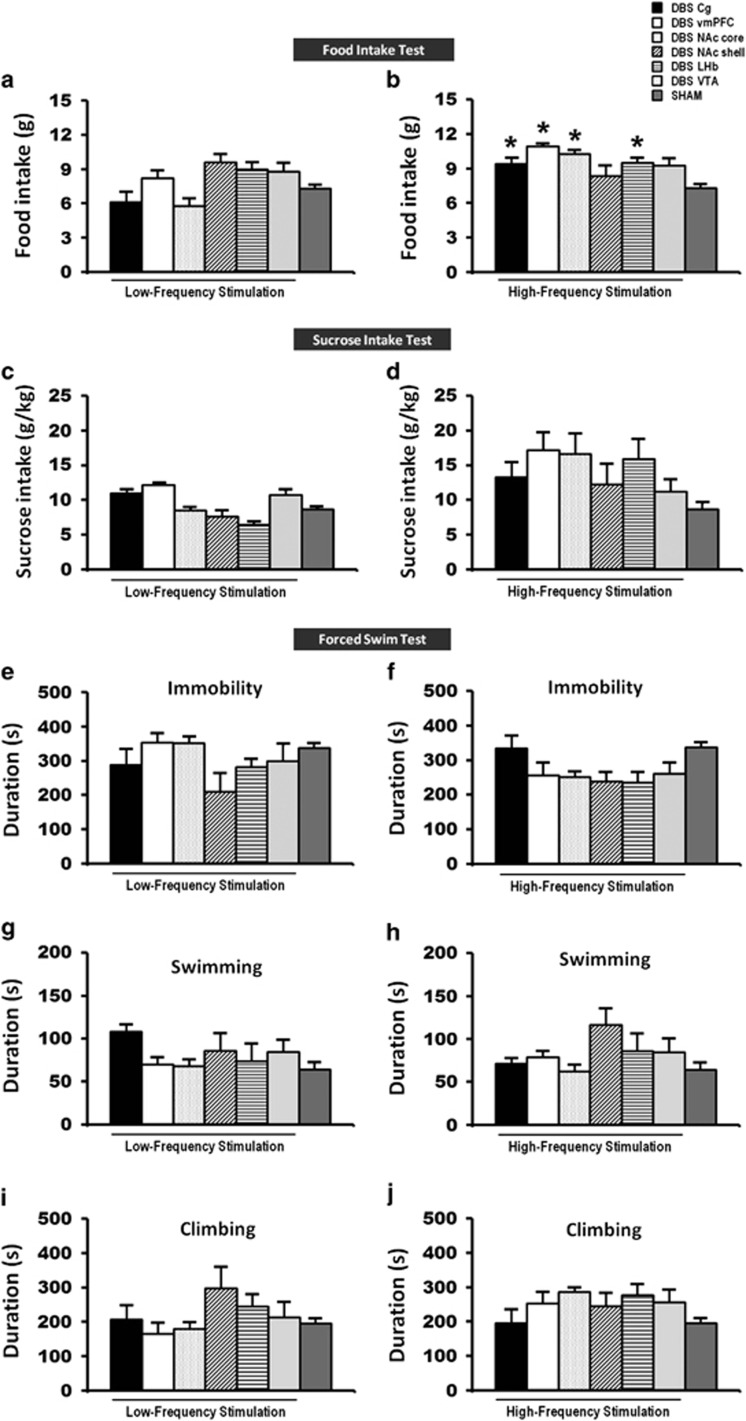
A set of bar graphs showing the measures of food motivation by food intake test (**a** and **b**), levels of anhedonia by sucrose intake test (**c** and **d**) and forced-swim behavior test (**e**–**j**) in naive animal experiments. Before testing, animals were stimulated for 15 min and continuously for 2 h in the food intake test, 1 h in the sucrose intake test and 10 min in the forced-swim test. HFS of the Cg, vmPFC, NAc core and LHb significantly increased the levels of motivation for food intake. No significant difference was found in the sucrose intake and forced-swim behaviors of both LFS and HFS groups, as well as the food intake test of LFS group. Data represent means±s.e.m. Significant difference from the sham animals, (^*^P<0.05). Cg, cingulate cortex; HFS, high-frequency stimulation; LFS, low-frequency stimulation; LHb, lateral habenular nucleus; NAc, nucleus accumbens; vmPFC, ventromedial prefrontal cortex.

Stimulation of the vmPFC,^11, 13, 14, 40^ NAc,^12^ ventral striatum,^16^ LHb^17, 18^ and VTA^19^ has previously been shown to contribute to antidepressant effects. We set out to determine which brain areas and which stimulation parameters resulted in the most optimal behavioral outcome following DBS. Our findings in the naive animal study show that HFS of the Cg, vmPFC, NAc core and LHb, but not of the NAc shell or of the VTA, resulted in a remarkable reduction of the escape latency time (F_(6,59)_=6.415, *P*<0.001; Figure 2b) in the home-cage emergence test, and furthermore resulted in a significant increase in food intake (F_(6,62)_=8.550, *P*=0.001; Figure 3b) as compared with sham animals. The Bonferroni *post hoc* tests for multiple comparisons showed that both LFS and HFS did not affect the behaviors of naive animals in the open-field center zone (LFS: F_(6,50)_=6.220, *P*=0.001; HFS: F_(6,52)_=2.755, *P*=0.021; Figures 2c and d), sucrose intake (LFS: F_(6,58)_=1.028, *P*=not significant (NS); HFS: F_(6,55)_=2.584, *P*=0.028; Figures 3c and d), nor any alternations in the duration of immobility (LFS: F_(6,52)_=2.156, *P*=NS; HFS: F_(6,51)_=3.388, *P*=0.007; Figures 3e and f), swimming (LFS: F_(6,52)_=1.174, *P*=NS; HFS: F_(6,51)_=2.059, *P*=NS; Figures 3g and h) and climbing (LFS: F_(6,52)_=1.419, *P*=NS; HFS: F_(6,51)_=1.792, *P*=NS; Figures 3i and j) activity in the forced-swim test. For locomotion behavior, we tested whether DBS might alter the locomotor activity and thereby confound the results of the behavioral tests. In the open-field, HFS of the VTA (F_(6,55)_=2.537, *P*=0.031) increased the motor parameter of distance moved. No difference was found on the open-field distance moved for LFS (F_(6,54)_=3.169, *P*=0.010).

In the sham implanted animals, there were no significant differences in the behaviors with respect to the implantation site (home-cage emergence test, F_(5,14)_=0.904, *P*=NS; open-field test, all variables F_(5,9)_>0.356, *P*=NS; food intake test, F_(5,16)_=1.358, *P*=NS; sucrose intake test, F_(5,15)_=0.298, *P*=NS; and forced-swim immobility test, F_(5,13)_=1.408, *P*=NS), and therefore we pooled all the animals into one sham group.

For the results of the comparison of the behavioral effects of DBS compared with the respective individual sham groups in naive animal experiment, please see Supplementary Table 1.

Summary of the behavior: HFS of the Cg, vmPFC, NAc core and LHb consistently resulted in reduced anxiety with decreased escape latency in the home-cage emergence test and increased motivation for food intake. No significance effects were found in the open-field, sucrose intake and forced-swim tests. HFS resulted in better behavioral outputs than LFS.

#### Behavioral effects of HFS in the CUS model

**Figure 4:**
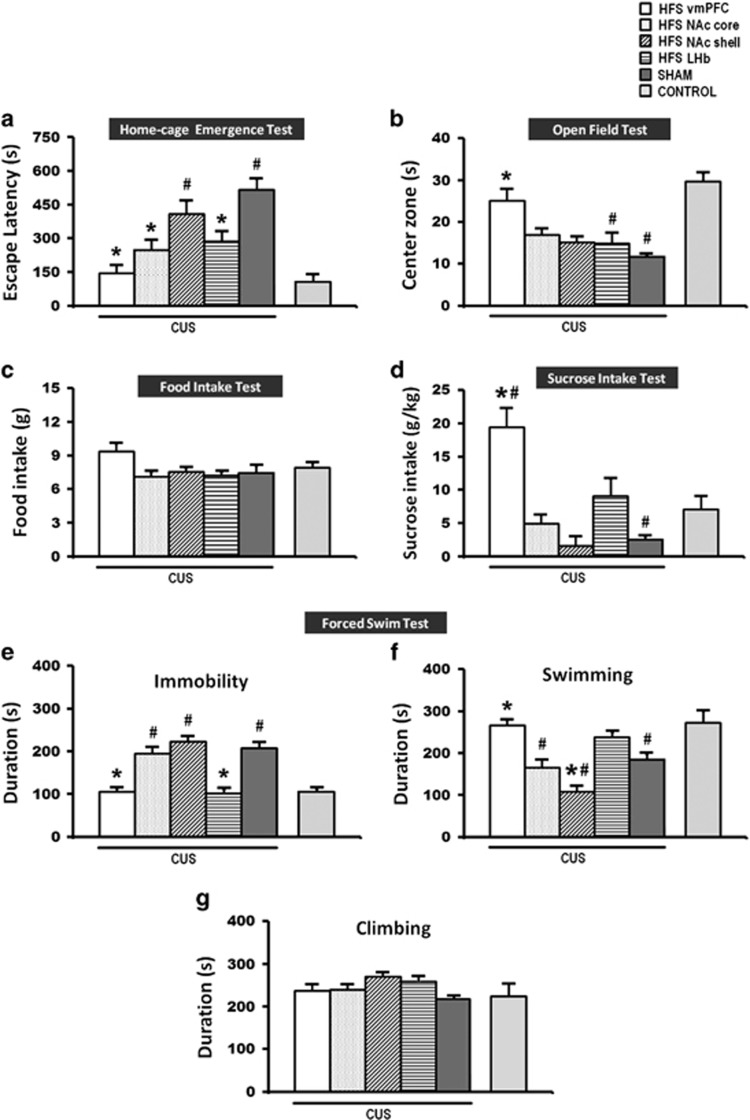
A set of bar graphs showing the measures of anxiety-like behavior by home-cage emergence (**a**) and open-field (**b**) tests, food motivation by food intake test (**c**), measures of anhedonia by sucrose intake test (**d**) and the forced-swim behavior test (**e**–**g**) after HFS in the CUS rat model of depression. Animals were similarly stimulated and tested as in the naive animal experiment. Note: HFS of the vmPFC, NAc core, and the LHb reduced anxiety-like behavior in the home-cage emergence test. However, in the open-field and sucrose intake tests, vmPFC HFS, but not other DBS targets, significantly increased time spent of rats in the open-field center zone and increased sucrose intake, indicating anxiolytic and alleviation of anhedonic-like behavior as compared with CUS sham rats. Finally, both the LHb and vmPFC HFS reduced forced-swim immobility. Data represent means±s.e.m. Significant difference from the CUS sham animals, ^*^P<0.05; significant difference from the non-CUS control animals, ^#^*P*<0.05. CUS, chronic unpredictable stress; DBS, deep brain stimulation; HFS, high-frequency stimulation; LHb, lateral habenular nucleus; NAc, nucleus accumbens; vmPFC, ventromedial prefrontal cortex.

To validate our findings from the naive animal study, we further tested our hypothesis that HFS of the vmPFC, NAC core (including NAc shell for comparison) and LHb on the behaviors of the CUS animal model. The same five behavioral paradigms as described for the previous experiment were again used here. In the CUS animal model, we found a significant phenotype difference for depressive-like behaviors between sham-CUS and non-CUS animals. Student *t*-tests revealed that CUS sham remarkably increased the escape latency from the home-cage (*t*_16_= 5.726, *P*<0.001; Figure 4a), decreased the time spent in the open-field center zone (*t*_17_=−4.198, *P*=0.001; Figure 4b), decreased sucrose intake (*t*_17_=−2.766, *P*=0.013; Figure 4d), increased immobility (*t*_20_= 6.790, *P*<0.001; Figure 4e) and decreased swimming time (*t*_17_=−3.120, *P*=0.005; Figure 4f) in the forced-swim test, as compared with control non-CUS animals. However, no effect was found for food intake (*t*_15_=−0.522, *P*=NS; Figure 4c). In terms of locomotor activity, no significant difference was found for the open-field distance moved (*t*_17_=−1.499, *P*=NS) and climbing (*t*_19_=−0.294, *P*=NS; Figure 4g) behavior in the forced-swim test.

In the home-cage emergence test, HFS of the vmPFC, NAc core and LHb reduced the escape latency of rats from their home-cage (F_(5,70)_=9.185, *P*=0.001; Figure 4a). Interestingly, HFS of the vmPFC, but not other DBS targets, resulted in significantly increased time spent by rats in the center zone of the open-field (F_(5,70)_=4.222, *P*=0.002; Figure 4b) and increased sucrose consumption substantially (F_(5,66)_=12.121, *P*=0.001; Figure 4d) when compared with the CUS sham animals. No behavioral alternation was found in the motivation for food intake (F_(5,64)_=2.914, *P*=0.02; Figure 4c). Strikingly, HFS of the vmPFC and the LHb significantly reduced the immobility time (F_(5,76)_=26.077, *P*<0.001; Figure 4e). There was also a significant effect of increased swimming time (F_(5,75)_=16.091, *P*<0.001; Figure 4f) found with vmPFC HFS. No significant changes were found in climbing time (F_(5,75)_=1.884, *P*=NS; Figure 4e). For locomotion behavior, NAc shell HFS significantly increased the distance moved (F_(5,70)_=5.217, *P*<0.001) in the open-field as compared with the CUS sham.

As for the CUS sham animals, no significant differences were found in the behaviors with respect to the implantation site (home-cage emergence test, F_(3,8)_=0.619, *P*=NS; open-field test, all variables F_(3,9)_>0.301, *P*=NS; food intake test, F_(3,6)_=0.917, *P*=NS; sucrose intake test, F_(3,8)_=0.566, *P*=NS; and forced-swim immobility test, F_(3,10)_=1.227, *P*=NS). Therefore, all CUS sham animals were pooled into one sham group.

For the results of the comparison of the behavioral effects of DBS compared with the respective individual sham groups in CUS animal experiment, please see Supplementary Table 2.

Summary of the behavior: In the CUS animal model, HFS of the vmPFC, NAc core and LHb resulted in anxiolytic effects in the home-cage emergence test. However, in the open-field and sucrose intake tests, vmPFC HFS consistently showed significant reduced anxiety and increased sucrose consumption in a comparison of all stimulated targets. In the forced-swim test, HFS of the vmPFC and LHb produced a drastic reduction in the duration of immobility. No difference was shown in the food intake test.

### Study 2

As HFS of the vmPFC resulted in the best antidepressant effects (with reduced anxiety levels in both the home-cage emergence and open-field tests, enhanced hedonia in sucrose intake test and reduction of forced-swim immobility) in the CUS animal model, we continued with only HFS of the vmPFC and investigated the 5-HT neuronal firing and morphology in the DRN. In addition, we also investigated the neural circuitry influenced by HFS of the vmPFC using c-Fos neuronal activation mapping approach in brain regions that have been implicated in the pathophysiology of depression in the CUS animal model.

#### Electrophysiological effects of vmPFC HFS on DRN 5-HT and non-5-HT neurons

**Figure 5:**
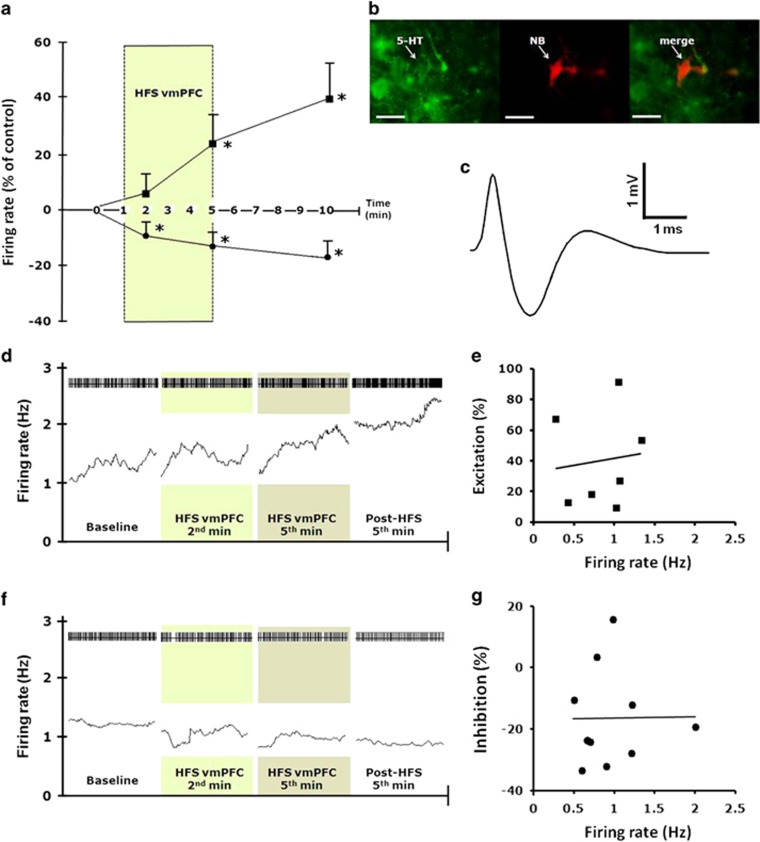
Effects of vmPFC HFS on DRN neuronal firing. (**a**) Excitatory and inhibitory effects on putative 5-HT DRN neurons (*n*=18) firing rate changes before, during and after vmPFC HFS. Darkened zone area indicates stimulation period (100 Hz, 100 μA, 100 μs). Firing rate was quantified during the final 60 s before stimulation as control baseline, during the second and fifth minute of vmPFC HFS, and on the fifth minute following cessation of vmPFC stimulation. Note, both excitatory and inhibitory effects on the firing rates of 5-HT neurons were observed by vmPFC HFS. (**b**) Photomicrographs showing immunohistochemical characterization of a neurobiotin (NB) juxtacellular-labeled 5-HT immunopositive neuron in the rat DRN. Scale bar, 20 μm at × 40 magnification. (**c**) A triphasic extracellular waveform shape with a broad spike width typically used to indicate for 5-HT neuron. (**d** and **f**) Spike trains and mean firing rate (15-s bins) of 5-HT putative neurons, before, during and after 5 min HFS of the vmPFC. Darkened zone areas indicate stimulation period (second and fifth minute of vmPFC HFS). Plot of percentage excitation (**e**) and inhibition (**g**) during the fifth minute after vmPFC HFS against baseline firing rates. Significant difference from the baseline levels ^*^P<0.05. 5-HT, 5-hydroxytryptamine, serotonin; DRN, dorsal raphe nucleus; HFS, high-frequency stimulation; vmPFC, ventromedial prefrontal cortex.

A total of 34 slow- and fast-firing DRN neurons were recorded (*n*=12 rats). Eighteen neurons had typical electrophysiological properties of 5-HT neurons: a slow firing rate (0.94±0.1 Hz), regular firing pattern (coefficient of variation, 0.32±0.02) and a triphasic waveform of wide spike duration (2.03±0.06 ms; Figure 5c). Sixteen neurons were fast-firing (2.96±1.0 Hz), had an irregular firing pattern (coefficient of variation, 0.86±0.1) and a shorter spike duration (1.64±0.12 ms). These are considered to be non-5-HT neurons.

HFS of the vmPFC increased the firing rate in seven putative 5-HT neurons and decreased it in 11 neurons. The effect was already present in the first 2 min, continued after cessation of HFS and increased over time (F_(3,27)_=5.232, *P*<0.01; Figures 5a and d). The activated 5-HT neurons (*n*=7) showed a mean increase in the firing rate during the second minute of 4.92±6.65%, during the fifth minute of 24.28±8.92% and after 5 min of 40.36±11.81%. In comparison, the inhibited 5-HT neurons (*n*=11, F_(3,43)_=4.122, *P*<0.01, Figures 5a and f) had a mean decrease of the firing rate of −10.98±3.25% and −12.71±3.21% during the second and fifth minute of HFS, respectively. After 5 min, the inhibition continued and was −15.05±4.72%. No significant correlation was found between baseline firing rate and the magnitude of the excitation and inhibition in neuronal firing (Figures 5e and g).

HFS of the vmPFC had no statistically significant effect on the firing rates of excited (*n*=5 neurons; F_(3,19)_=1.504, *P*=0.25) and nonresponding non 5-HT cells (*n*=8 neurons; F_(3,31)_=0.848, *P*=0.48). However, there was a significant effect in the firing rate of non 5-HT neurons that showed inhibition (*n*=3 neurons; F_(3,11)_=7.611, *P*<0.01) with a mean decrease of the firing rate during the second minute of −8.40±2.16%, during the fifth minute of −19.27±2.52%, and after 5 min of −20.04±6.09%.

#### Histochemical effects of vmPFC HFS on DRN neurons

**Figure 6:**
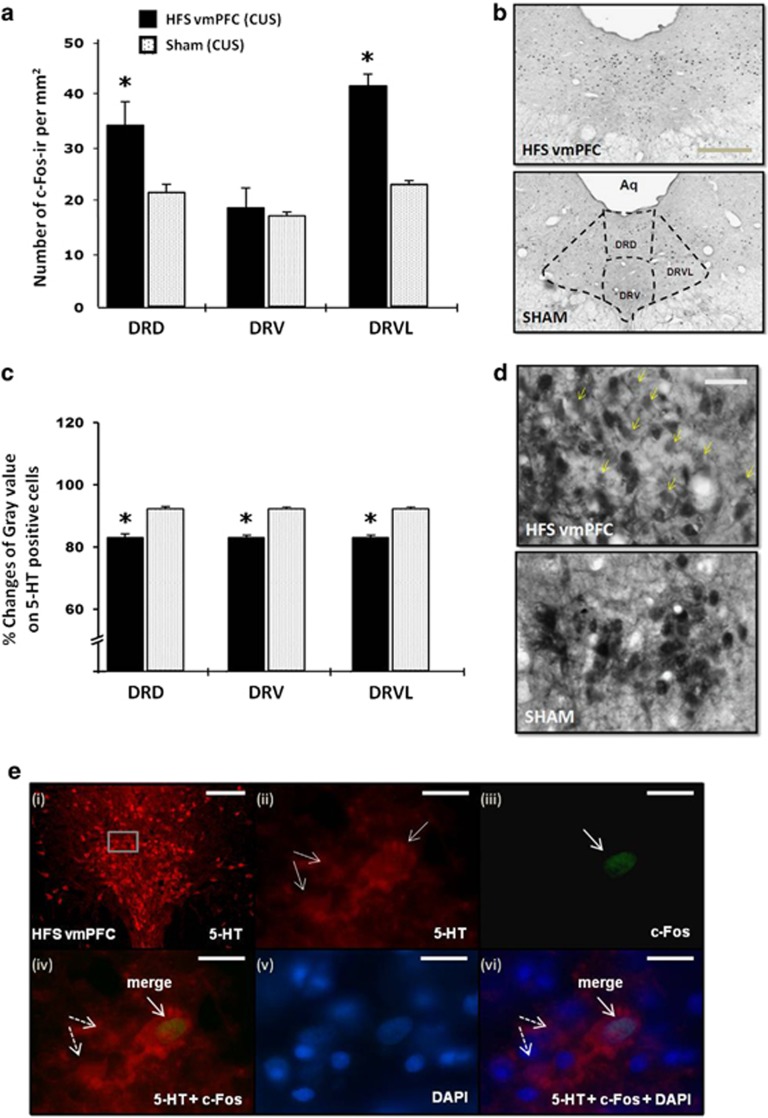
Effects of vmPFC HFS on the c-Fos neural activity (**a**) and the percentage changes of mean gray value on 5-HT positive cells (**c**) in the DRN region of CUS animal model of depression. Representative photomicrographs of c-Fos (**b**; scale bar, 500 μm at × 4 magnification) and 5-HT (**d**; scale bar, 50 μm at × 40 magnification) histochemical staining of 30-μm-thick sections in the DRN. Note, vmPFC HFS resulted in higher levels of neuronal activation (that is, increased c-Fos positive cells) in the DRN areas as compared with the CUS sham animals. The small dots represent c-Fos-ir cells per mm^2^. Meanwhile, vmPFC HFS caused a remarkable reduction of optical density of 5-HT containing cells in the DRN area, indicating a local release of 5-HT, which increases in extracellular 5-HT in the DRN and forebrain projection areas. Representative high-power confocal images (**e**) are shown for the localization of 5-HT (red; **e**-**i** and -**ii**) and c-Fos (green; **e**-**iii**) immunofluorescence positive cells. DAPI (blue; **e**-**v**) was used as a nuclear stain. Merged images demonstrated the co-localization (arrows) of 5-HT and c-Fos (**e**-**iv**), as well as counterstained with DAPI (**e**-**vi**) in the DRD. Dotted arrows demonstrated non-c-Fos 5-HT positive cells (**e**-**iv** and -**vi**). Scale bar for fluorescent images: 100 μm (**e**-**i**) at low-power magnification; and 20 μm (**e**-**ii**–**vi**) at × 100 magnification. Significant difference from the CUS sham animals, ^*^P<0.05. 5-HT, 5-hydroxytryptamine, serotonin; Aq, aqueduct; CUS, chronic unpredictable stress; DAPI, 4',6-diamidino-2-phenylindole; DRN, dorsal raphe nucleus dorsal part; DRV, dorsal raphe nucleus ventral part; DRVL, dorsal raphe nucleus ventrolateral part; HFS, high-frequency stimulation; vmPFC, ventromedial prefrontal cortex.

HFS of the vmPFC significantly increased the number of c-Fos-ir cells in the median (dorsal raphe dorsal part, DRD (*t*_10_=2.459, *P*=0.034)) and lateral wings (dorsal raphe ventrolateral part, DRVL (*t*_9_=9.068, *P*=0.001)) of DRN regions as compared with CUS sham animals, indicating a modulation of the DRN 5-HT (median part) and non 5-HT neurons (lateral wings) in response to vmPFC HFS (Figures 6a and b). No significant difference was found in the dorsal raphe ventral part, DRV (*t*_10_=0.416, *P*=0.686). This is in line with our electrophysiology data. Further evaluation of the 5-HT-containing cells revealed that vmPFC HFS significantly lowered the optical density signal in the 5-HT cells of the DRD, DRV and DRVL (all *t*<−8.230, *P*<0.001) as compared with the CUS sham group (Figures 6c and d). To further characterize the nature of the investigated cells, qualitative assessment of the immunofluorescence double-labeled sections showed co-localization between 5-HT and c-Fos in the DRD region (Figure 6e).

#### Effects of vmPFC HFS in the neural circuitry of depressive-like behavior

Using a neuronal mapping approach, we investigated the target areas of the DRN 5-HT system in the forebrain (see Table 1). In the frontal cortex regions, we found enhanced levels of c-Fos-ir cells in the prelimbic cortex (*U*=6, *P*=0.032), except for the cingulate cortex area 1 and 2 (all *U*<16, *P*>0.253), ventral orbital cortex (*t*_11_=−0.009, *P*=0.993), lateral orbital cortex (*t*_11_=0.656, *P*=0.525), and infralimbic cortex (*U*=11, *P*=0.571). No significant effects were found in both the NAc core and shell parts (all *U*<19, *P*>0.475). In the habenula, there was an increase of c-Fos-ir cells in the lateral part (*t*_10_=2.573, *P*=0.042), and not the medial part of habenular nucleus (*U*=12, *P*=0.584). In the hippocampus, activation of c-Fos-ir cells was shown in the hippocampal CA1 (*t*_10_=2.573, *P*=0.028) and CA3 (*t*_10_=2.483, *P*=0.032) fields, but not the hippocampal dentate gyrus (*t*_9_=0.047, *P*=0.964). In the amygdala, vmPFC HFS specifically activated the c-Fos-ir expression in the lateral (*t*_10_=2.772, *P*=0.020) and basolateral (*t*_10_=2.269, *P*=0.047) amygdaloid nucleus. No difference was detected in the medial (*t*_10_=0.361, *P*=0.725) and central (*t*_10_=1.732, *P*=0.114) amygdaloid nucleus. In the hypothalamus, there was an activation of c-Fos-ir expression in the anterior (*t*_8_=3.258, *P*=0.012) and dorsomedial (*t*_8_=2.314, *P*=0.049) hypothalamus, and the medial parvicellular part of the paraventricular hypothalamic nucleus (*t*_9_=2.569, *P*=0.030). However, there was no significant change of c-Fos expression found in the posterior (*t*_10_=1.571, *P*=0.147), medial (*t*_10_=0.804, *P*=0.440) and perifornical nucleus of the hypothalamus (*t*_9_=1.517, *P*=0.164), as well as the lateral magnocellular part of the paraventricular hypothalamic nucleus (*t*_8_=1.305, *P*=0.228). In the periaqueductal gray, no significant difference was found in all regions of both the anterior and posterior parts (*t*_11_>−0.574, *P*>0.274).

Interestingly, we found a decrease of c-Fos expression in the deep cerebellar nucleus of the magnocelluar part of the interposed cebebellar nucleus (*t*_10_=−2.234, *P*=0.049). No significant differences were found in the dentate nucleus of cerebellum (*t*_11_=−1.175, *P*=0.265), parvicelluar part of the interposed cebebellar nucleus (*t*_11_=−0.427, *P*=0.678), fastigial cerebellar nucleus (*t*_11_=−1.180, *P*=0.263), parvicellular part of the medial vestibular nucleus (*t*_11_=−0.894, *P*=0.391), magnocellular part of the medial vestibular nucleus (*t*_11_=−0.798, *P*=0.442) and the spinal vestibular nucleus (*t*_11_=−0.935, *P*=0.370).

## Discussion

DBS is a potential therapy for patients with major depression. Different brain targets have been explored and different outcomes have been obtained with respect to depressive-like symptoms. Here, we compared the depressive-like behavioral outputs of DBS of different brain areas in naive animals and a model of depression. Our results in the naive animals revealed that HFS, but not LFS, of the Cg, vmPFC, NAc core and LHb reduced anxiety levels, as indicated by shorter escape latency and an increased motivation for food consumption. No significant difference of DBS was detected in the open-field, sucrose intake and forced-swim tests, as compared with the sham.

The features of clinical depression include symptoms of emotional instability (low mood, anxiety), anhedonia, poor motivation, fatigue and hopelessness.^22^ Our present CUS model produced a robust behavioral phenotype. After 3 weeks of consecutive stress, animals displayed elevated levels of anxiety, increased latency when escaping from the home-cage and decreased time spent in the open-field center zone when compared with nonstressed controls. In addition, there was also a decrease of sucrose consumption and an increased immobility time in the forced-swim test of the CUS sham animals, suggesting anhedonia and behavioral despair.^41^ Our results show that HFS of the vmPFC, NAc Core and LHb reduced anxiety in the home-cage emergence test. However, in the open-field and sucrose intake tests, only vmPFC HFS, but not other targets, resulted in major improvements in terms of anxiety and increased sucrose consumption, indicating a reduction of anhedonic-like behavior, one of the core symptoms of depression.^42, 43^ In the forced-swim test, HFS of the vmPFC and LHb significantly reduced immobility time as compared with CUS sham. Taken together, our behavioral findings identify the vmPFC as a specific and potent structure to relieve depressive-like symptoms by HFS when compared with other stimulated targets. HFS of the NAc core and LHb showed partial effects on the depressive-like behavioral outputs.

The present DBS study in naive animals was used to determine which brain targets and which stimulation paradigms (either LFS or HFS) were the most effective for antidepressant activities. Our data clearly showed that HFS of the Cg, vmPFC, NAc core and LHb results in improvements to both anxiety and motivation for food intake. However, no changes in DBS effects were found in the open-field, sucrose intake and forced-swim tests. These findings were partially consistent with the results of Hamani *et al.*,^44^ which showed that HFS (at amplitude 100 and 200 μA), but not LFS of the vmPFC induced antidepressant response in the forced-swim test of naive animals. Although we found no difference in terms of stimulation effects on the forced-swim behaviors, it is important to highlight here that we compared the effects across different brain targets using the more stringent Bonferroni *post hoc* test to avoid false positives. In addition, when the individual DBS target(s) in our study was analyzed and compared with sham animals independently, there were significant stimulation effects found on the behavioral paradigms (see Supplementary Tables 1 and 2). However, the main purpose of our study was to compare across different DBS targets and determine which brain target(s) elicited the most optimal behavioral outcomes. Thus, the statistical analyses were conducted using Bonferroni *post hoc* tests to provide adequate protection against increased error rates in multiple comparisons. An alternative explanation for the nonsignificant effects found in the open-field, sucrose intake and forced-swim tests, is that they could be due to a nonpathological depressive condition in naive animals.^36^ As our results clearly demonstrated a significance difference of behavioral phenotypes between CUS and non-CUS naive animals, we therefore tested our hypothesis in this validated CUS animal model^35, 36^ using previously determined brain targets and stimulation parameters that were derived from the naive animal study.

Interestingly, in a comparison of all stimulated targets, we found that HFS of the vmPFC produced the most profound antidepressant effects with reduced anxiety, decreased anhedonia and forced-swim immobility in the CUS model. Although HFS of the LHb counteracted the CUS-induced changes in the home-cage emergence escape latency and forced-swim immobility, NAc core HFS only normalized its escape latency. To evaluate the physiological impact of DBS on these targets, it has been shown that vmPFC HFS (at 100/200 μA, 130 Hz and 90 μs) increased the hippocampal 5-HT release (fourfold increase) and brain-derived neurotrophic factor levels in the CUS rats,^13, 14^ with significant contribution to antidepressant-like effects. Similarly, LHb stimulation (at 15 Hz, 500 μA) produced a 55–70% increase in striatal 5-HT release, and its stimulation at 1.5–3 Hz induced no detectable changes in striatal extracellular 5-HT levels.^45^ Further, stimulation (at 100 μA, 130 Hz and 100 μs) of the dorsal–ventral striatum (NAc core), but not ventral–ventral striatum (part of NAc shell) increased neuronal brain-derived neurotrophic factor in the mPFC region.^46^ A further study also showed that NAc core HFS (at 300 μA, 120 Hz and 80 μs) enhanced 5-HT levels in the mPFC with ~27% increase compared with baseline.^47^ In contrast, our previous findings found no stimulation effects on the 5-HT levels in the mPFC structure with respect to both NAc core and NAc shell stimulation (at 30 μA, 130 Hz and 60 μs),^48^ and we also demonstrated that NAc shell, but not NAc core, HFS increased 5-HT levels in the NAc region. Despite these controversial findings on NAc stimulation, the effects of DBS depend largely on specific stimulation parameters, in which HFS and optimum amplitude have an important role for antidepressant-like effects. In the present study, although we did not examine the 5-HT levels in our comparison of these stimulated targets, it is still worth noting that vmPFC HFS was found to be the best target for antidepressant effects behaviorally. This notion is highly supported by previous studies, which show that vmPFC HFS produced fourfold of hippocampal 5-HT extracellular release, while LHb and NAc core stimulation produced ~55–70% and 27% increase of 5-HT levels in the forebrain region as compared with baseline, respectively.^45, 47^

As vmPFC HFS has been identified as the most optimal target for antidepressant-like response, we further looked into its functional role in the neurocircuitry of depressive-like behavior. The vmPFC has a pivotal role in modulating sadness and negative emotions in both depressed and healthy subjects.^8, 49^ It has predominant reciprocal connections with the limbic brain areas that are involved in emotions and reward processes, including the amygdala, hippocampus, hypothalamus and midbrain.^50, 51^ In this study, HFS of the vmPFC evoked a widespread, but selective, modulation of these brain areas. Our anatomical mapping study showed that HFS of the vmPFC modulated the cellular activity in specific areas within the vmPFC (prelimbic area), LHb, hippocampus (CA1 and CA3 regions), lateral and basolateral amygdaloid nucleus, anterior and dorsomedial hypothalamus and the medial parvicellular part of the paraventricular hypothalamic nucleus. There was also a decreased c-Fos expression in the deep cerebellar nucleus of the magnocelluar part of the interposed cerebellar nucleus. Interestingly, no significant difference was found in the periaqueductal gray, a region known to be involved in the expression of fear and defensive behavior.^25^ Our findings suggest that HFS of the vmPFC modulated a brain circuit, which is crucial for the regulation of emotions implicated in depression. This is in line with clinical studies of individuals with depression showing changes of brain volume and function in the amygdala, hippocampus and PFC.^52, 53^ Converging evidence from clinical and animal studies have also demonstrated that depression is not a single brain region disorder, but a multisystem disorder affecting the cortico–limbic pathway as well as the midbrain regions. For review, see Franklin *et al.*^54^

In clinical research, functional neuroimaging studies have demonstrated that hypoactivation observed in the vmPFC is closely associated with patients suffering from posttraumatic stress disorder.^55, 56^ Similarly, such hypoactivity or abnormality in the subgenual prefrontal cortex has also been found in depressed patients^53, 57^ or individuals with childhood emotional maltreatment.^58^ This is consistent with animal models showing lower neural activity or decreased immediate early gene expression in the mPFC structure following stress exposure (for instance social defeat, predator stress or water submersion).^11, 59^ In the present study, we showed the beneficial effect of antidepressant activity with reversal of this hypoactivity by HFS of the vmPFC. In line with our observation, various methods of stimulation by optogenetic or transcranial magnetic stimulation have also induced significant improvement on behavioral outcome by rescuing the hypofunction of the vmPFC.^60, 61^ Importantly, DBS induced striking changes in these brain regions with reversal of abnormal cerebral blood flow and glucose metabolism leading to sustained mood improvement in case series of depressed patients.^8^

The vmPFC, including ventral prelimbic and infralimbic regions, regulates not only emotion and memory functions,^62^ but also autonomic and neuroendocrine responses in stressful conditions.^63, 64^ In anatomical and functional connectivity, the vmPFC has reciprocal connections with the hypothalamus, which mediates the neuroendocrine function in response to stress through activation of the hypothalamic–pituitary–adrenal axis by stimulation of the neurons in the medial parvicellular part of the paraventricular hypothalamic nucleus.^54^ In addition, the hippocampus also has direct or indirect projections to the paraventricular hypothalamic nucleus, which negatively regulates the hypothalamic–pituitary–adrenal axis function. In this study, vmPFC HFS induced remarkable c-Fos neuronal activity within the hippocampal regions and paraventricular hypothalamic nucleus, indicating a regulatory role of the vmPFC in modulating stress response through the hippocampus–hypothalamic–pituitary–adrenal axis pathway. The connection of vmPFC–hippocampus is mainly by way of direct projection from the hippocampal CA1 and subiculum to the prelimbic, infralimbic and medial orbital areas.^65^ On the other hand, the projection of vmPFC to the hippocampus is via the nucleus reunions that function as a relay for the convergence of limbic-related information from widespread regions.^66^ This implies an involvement of the limbic–thalamo–cortical circuits in emotional control for depression, as previously proposed.^57, 67^

In the cerebellum, it has been demonstrated that patients with cognitive impairments had reduced metabolic activity (cerebral blood flow) in the vmPFC and increased activity in the cerebellum.^68^ Functional imaging studies also showed that the increased activity in the cerebellum is highly correlated with emotional instability such as sadness, feeling empathy and viewing emotional pictures.^69, 70^ Importantly, the cerebellum has robust anatomical connections to the limbic system.^71, 72, 73^ One recent study using retrograde transneuronal transport of the rabies virus has identified a connection of the deep cerebellar nuclei—more specifically, the interpositus nucleus projects to the prefrontal cortex via the thalamus.^74^ In line with previous studies, our present results show decreased c-Fos expression in the magnocellular part of the interposed cerebellar nucleus by vmPFC HFS, suggesting a role for the cerebro–thalamo–cortical pathway.

The vmPFC has strong reciprocal connections with a wide range of structures including the amygdala, habenula and a number of brain stem nuclei implicated in the regulation of mood and anxiety disorders.^64^ Many studies have established a specific amygdala–frontal circuit for the control, expression and experience of negative emotions.^75, 76^ Furthermore, the interaction between vmPFC and LHb has imposed a primary cortico–hebenular pathway.^18, 77^ There is also strong evidence supporting the LHb–DRN interaction which assigns the LHb a major role in 5-HT regulation and its neurotransmitter release from the DRN to the forebrain.^78, 79^ The pattern of c-Fos expression in the present study showed that HFS of the vmPFC influences the neuronal circuit for depressive-like behaviors, which is likely to be mediated in the DRN. A potential mediator of this effect could be the midbrain 5-HT system, which is known to project to the key areas involved in emotions, as mentioned above.^80, 81^ With HFS of the vmPFC, we found a profound and specific modulation of DRN neurons, the major source of 5-HT to the forebrain, shown both electrophysiologically and histochemically. The neurochemical effect would be an enhanced release of 5-HT in the target areas. This has been previously demonstrated.^14, 44^

Although immediate early genes (such as c-Fos) are not a ubiquitous marker for studying connectivity, they have been widely used in DBS studies in animal models of mood and anxiety disorders.^28, 82^ In a recent study, vmPFC HFS induced a profound increase of Zif268 expression, another immediate early gene, in the prelimblic cortical region.^82^ This is in line with our findings.

Our electrophysiological recordings showed an effect of vmPFC stimulation on neuronal activity in the DRN. We found that vmPFC HFS induced either excitation (39%) or inhibition (61%) of cell firing in the 5-HT neurons tested. Meanwhile, non-5-HT neurons showed either inhibition (19%) or were nonresponsive to vmPFC HFS. The main findings were that vmPFC HFS evoked a specific modulation of both the 5-HT and non-5-HT cell firing. This is in accordance with previous evidence showing that DRN inputs from the prefrontal cortex are able to influence the neuronal firing rate by either directly activating the DRN 5-HT neurons or by local GABAergic interneurons.^81, 83^ Moreover, earlier work has also suggested reciprocal actions of 5-HT and GABA, demonstrating GABA inhibitory inputs to the DRN 5-HT neurons,^84, 85^ contributing to the inhibition of 5-HT cell firing.

The firing rate of a 5-HT neuron is commonly the determinant of 5-HT neurotransmission and function in a terminal region.^86^ It has been shown that bilateral subthalamic nucleus HFS caused a moderate inhibition of 5-HT cell firing in the DRN, which evoked clear-cut, depressive-like behavior in the forced-swim test.^29^ In the present study, one would assume that this inhibition of 5-HT cell firing might possibly be linked to depressive-like effects, as in the case of subthalamic nuclear stimulation. Nevertheless, it is important to note that the behavioral effects obtained from subthalamic nucleus and vmPFC HFS were completely opposite to this. Although we showed ~61% inhibition of cell firing in the 5-HT neurons with vmPFC HFS, our stimulation elicited antidepressant activity. This is further supported by microdialysis studies in which subthalamic nucleus HFS reduced extracellular 5-HT,^87, 88^ whereas vmPFC HFS enhanced its 5-HT release in specific forebrain regions.^14, 44, 89^ One plausible explanation of the inhibitory effect is that it is due to the mechanisms of autoinhibition of 5-HT cells caused by endogenous local release of 5-HT from activated or neighboring cells.^90, 91^ This could also be achieved by 5-HT_1A_ autoreceptor-mediated feedback inhibition of 5-HT cells that are found on the dendrites and cell bodies of these neurons.^90, 92^ Another possible explanation is that such an inhibitory effect on 5-HT firing rate can result from GABA–5-HT interaction. This has been demonstrated by intra-DRN administration of GABA antagonists that enhanced 5-HT cell firing and release,^93, 94^ and eventually inhibited 5-HT cell firing by the autoinhibition mechanism.

In conclusion, we found that HFS of the vmPFC produced promising antidepressant effects with enhanced hedonia and decreased anxiety and forced-swim immobility in the CUS animal model. This effect was accompanied by modulation of a number of key regions involved in emotional processing by a DRN-dependent mechanism. Overall, our findings identify the vmPFC as a specific and potent modulator of depressive-like behaviors, and as a favorable target for DBS in refractory depression.

## Figures and Tables

**Table 1:** Effects of vmPFC HFS on c-Fos-ir expression in brain regions implicated in the pathophysiology of depression

*Brain regions*	*Subregion*	*HFS vmPFC (CUS)*		*SHAM (CUS)*		*P-value*
		*Mean*	*±SEM*	*Mean*	*±SEM*	
**Frontal cortex**	**Cg1**	162.90	71.98	78.67	21.85	*P*=0.475
	**Cg 2**	162.09	92.83	52.07	14.79	*P*=0.253
	**VO**	380.93	87.34	382.16	102.36	*P*=0.993
	**LO**	449.34	75.89	345.48	147.13	*P*=0.525
	**PrL**	267.64	109.68	94.11	18.07	***P*****=0.032**
	**IL**	146.35	85.79	87.22	25.37	*P*=0.571
**Nucleus accumbens**	**NAc core**	80.57	44.03	36.13	10.99	*P*=0.475
	**NAc shell**	106.16	63.94	53.73	12.60	*P*=0.775
**Habenula**	**MHb**	3.93	0.93	6.07	3.41	*P*=0.584
	**LHb**	68.94	18.24	15.87	9.62	***P*****=0.042**
**Hippocampus**	**CA1**	199.25	52.31	50.78	24.37	***P*****=0.028**
	**CA3**	146.70	25.19	59.25	24.60	***P*****=0.032**
	**DG**	200.23	42.12	196.32	77.25	*P*=0.964
**Amygdala**	**MeA**	153.95	31.92	140.90	16.88	*P*=0.725
	**CeA**	108.66	25.90	56.48	15.39	*P*=0.114
	**LA**	168.26	24.94	78.35	20.74	***P*****=0.020**
	**BLA**	113.93	12.61	76.02	10.97	***P*****=0.047**
**Hypothalamus**	**DMH**	284.27	43.68	174.60	18.40	***P*****=0.049**
	**PeFLH**	205.34	25.82	161.05	6.83	*P*=0.164
	**AH**	100.23	18.96	34.36	7.02	***P*****=0.012**
	**PH**	264.27	27.73	191.70	36.94	*P*=0.147
	**VMH**	148.86	26.46	118.81	26.40	*P*=0.440
	**PaMP**	157.11	35.45	52.53	12.22	***P*****=0.030**
	**PaLM**	195.39	45.09	107.26	47.09	*P*=0.228
**Anterior periaqueductal gray**	**dmPAG**	78.26	9.30	67.60	18.23	*P*=0.597
	**dlPAG**	92.36	15.25	73.42	19.93	*P*=0.459
	**lPAG**	154.58	19.20	119.19	24.57	*P*=0.274
	**vlPAG**	127.01	19.51	114.97	38.24	*P*=0.775
**Posterior periaqueductal gray**	**dmPAG**	136.07	14.06	116.16	21.82	*P*=0.446
	**dlPAG**	111.43	12.95	110.03	22.74	*P*=0.956
	**lPAG**	142.78	11.66	146.59	27.23	*P*=0.894
	**vlPAG**	153.17	15.65	171.02	28.34	*P*=0.578
**Deep cerebellar nuclei**	**Dent**	92.03	13.79	107.68	3.76	*P*=0.265
	**IntMC**	81.56	6.05	118.03	12.95	***P*****=0.049**
	**IntPC**	113.39	28.10	126.04	13.37	*P*=0.678
	**Fast**	44.39	11.87	59.85	6.66	*P*=0.263
**Vestibular nuclei**	**MVePC**	20.70	3.38	26.00	4.65	*P*=0.391
	**MVeMC**	25.40	4.45	30.53	4.58	*P*=0.442
	**SpVe**	60.85	7.95	68.80	4.00	*P*=0.370
**Dorsal raphe nucleus**	**DRD**	34.11	4.94	21.74	2.06	***P*****=0.034**
	**DRV**	18.80	5.28	17.03	3.39	*P*=0.686
	**DRVL**	41.86	2.49	23.07	1.22	***P*****=0.001**

Abbreviations: AH, anterior hypothalamus; BLA, basolateral amygdaloid nucleus; CA1, hippocampal CA1 field; CA3, hippocampal CA3 field; CeA, central amygdaloid nucleus; Cg1, dorsal cingulate cortex area 1; Cg2, dorsal cingulate cortex area 2; Dent, dentate nucleus of cerebellum; DG, dentate gyrus; DMH, dorsomedial hypothalamus; dlPAG, dorsolateral periaqueductal gray; dmPAG, dorsomedial periaqueductal gray; DRD, dorsal raphe nucleus, dorsal part; DRV, dorsal raphe nucleus, ventral part; DRVL, dorsal raphe nucleus, ventrolateral part; Fast, fastigial cerebellar nucleus; IL, infralimbic cortex; IntMC, interposed cebebellar nucleus, magnocelluar part; IntPC, interposed cebebellar nucleus, parvicelluar part; LA, lateral amygdaloid nucleus; LHb, lateral habenular nucleus; LO, lateral orbital cortex; lPAG, lateral periaqueductal gray; MeA, medial amygdaloid nucleus; MHb, medial habenular nucleus; MVeMC, medial vestibular nucleus, magnocellular part; MVePC, medial vestibular nucleus, parvicellular part; NAc core, nucleus accumbens core; NAc shell, nucleus accumbens shell; PaLM, paraventricular hypothalamic nucleus, lateral magnocellular; PaMP, paraventricular hypothalamic nucleus, medial parvicellular; PeFLH, perifornical nucleus of the hypothalamus; PH, posterior hypothalamus; PrL, prelimbic cortex; SpVe, spinal vestibular nucleus; vlPAG, ventrolateral periaqueductal gray; VMH, ventromedial hypothalamus; VO, ventral orbital cortex. Before being killed, animals received 1 h stimulation and 1 h interval in their home-cage to determine the peak level of nuclear protein c-Fos-ir expression. Note, a remarkable increase of c-Fos neuronal activation was detected in the vmPFC (PrL), LHb, hippocampus (CA1, CA3), amygdala (LA, BLA), hypothalamus (DMH, AH, PaMP) and DRN (DRD, DRVL). Interestingly, there was a decrease of c-Fos expression found in the deep cerebellar nucleus (IntMC). Data represent means±s.e.m. of c-Fos-ir cells per mm^2^. P-value o0.05 (in bold) indicates statistically significant difference from the Sham CUS animals.
