# Supplementary material for: Electrical stimulation alleviates depressive-like behaviors of rats: investigation of brain targets and potential mechanisms
Source: Transl Psychiatry. 2015 Mar 31;5(3):e535–. doi: 10.1038/tp.2015.24 (PMC4354354; doi:10.1038/tp.2015.24)
Supplement: Supplementary Information [file tp201524x1.doc]

**Supplementary Methods**

**1. Surgical procedures**

*Surgery for behavioral experiments*

Rats were sedated throughout the entire procedure using 2.5% isoflurane inhalation anesthesia. Animals were placed in a stereotactic apparatus (Stoelting, Wood Dale, USA). After making a burr hole in the skull, they received implantation of the electrodes at the level of their respective coordinates according to the atlas of Paxinos & Watson. A construction of one gold-plated needle-like electrode with an inner wire of a platinum–iridium combination (Technomed, Beek, The Netherlands), with a tip diameter of 50 μm and a shaft diameter of 250 μm, was employed in this study. After implantation, the electrodes were secured to the skull in position using dental cement (Heraeus Kulzer, Hanau, Germany). Postoperatively, the animals had a two-week recovery period.

*Surgery for electrophysiological experiments*

Rats were anesthetized using chloral hydrate (460 mg/kg, i.p., J.M. Loveridge Ltd., Southampton, UK) and mounted in a stereotactic frame (David Kopf Instruments, Tujunga, CA, USA). Throughout the experiment, animals remained anesthetized to a deep surgical level, with additional doses of chloral hydrate as required. Body temperature was maintained at 36 ± 0.5 °C via a homoeothermic heating blanket connected to a rectal probe. General anesthesia was monitored using electrocorticograms (high frequency-pass filtered at 0.1 Hz with ×2000 amplification) recorded via steel skull screws located above the left prefrontal cortex, with the contralateral cerebellum serving as a reference.

After a burr hole was drilled, a glass recording microelectrode (12–25 MΩ impedance in vivo) filled with 0.5 M NaCl and neurobiotin (1.5% w/v; Vector Laboratories, Peterborough, UK) was descended into the DRN (coordinates (mm) from bregma: anteroposterior −7.5, mediolateral 0.0 and dorsoventral −4.5 to −5.5) using a piezoelectric microdrive (Inchworm, EXFO, Quebec, Canada). Mineral oil and saline solution (0.9% NaCl) were applied to the exposed cortex surface or skull areas to prevent exposed tissue from dehydration.

**2. Electrophysiological protocol**

*In-vivo extracellular recording DRN neurons*

Extracellular single-unit recordings of putative 5-HT were performed as previously described ^1^. Putative 5-HT neurons satisfied the following electrophysiological criteria: slow (<2.5 Hz) and regular (coefficient of variation, <0.5) firing pattern, triphasic extracellular spike waveform with a broad spike width (1.0–2.5 ms). DRN neurons that displayed a slow and regular firing rate but fired doublet or triplets of action potentials at high frequency (<20 ms; stereotyped burst firing), were also selected, as such neurons have been shown to contain 5-HT ^2, 3^. In comparison, putative non-5-HT neurons were identified as having a fast and irregular firing and less-broad spike duration.

After 5 min of baseline recording of individual DRN neurons, HFS vmPFC stimulations were performed for 5 min, and recordings continued for a further 5 min after the cessation of the stimulus. The same neuron was then subject to juxtacellular labeling for post-mortem neurochemical identification. Alternatively, after recording, the final position of the electrode was marked by iontophoretic dye injection.

Biopotentials were amplified (×10) through the active bridge circuitry of a Neurodata amplifier (Cygnus Technologies, Delaware Water Gap, PA,. USA), AC-coupled, and further amplified (×100; NL106 AC-DC Amp; NeuroLog System, Digitimer Ltd, Welwyn Garden City, Hertfordshire, UK), before being filtered between 0.3 and 5 kHz (NL125; NeuroLog System, Digitimer Ltd, Welwyn Garden City, Hertfordshire, UK). Signals were digitized online using a Micro1401 Analog-Digital converter (Cambridge Electronic Design, Cambridge, UK) and a PC running Spike2v6 acquisition and analysis software (Cambridge Electronic Design, Cambridge, UK). Single unit activity and electrocorticograms were sampled at 20 kHz.

*Stimulation electrodes and parameters*

Stimulation electrodes were stereotactically implanted bilaterally into the vmPFC (AP: + 2.70 mm; L: ± 0.60 mm; V: - 4.60 mm). The electrodes (concentric bipolar coaxial design) were gold-coated with an inner wire of platinum-iridium combination (tip diameter 50 μm, shaft diameter 250 μm; Technomed Europe, Beek, The Netherlands). Each electrode was connected to a constant-current stimulus isolation unit (A360, World Precision Instruments, Aston, Stevenage, UK), driven simultaneously by a Master 8 pulse generator (AMPI, Jerusalem, Israel). Stimulations parameters were: 100μA amplitude, 100Hz frequency, and 100μs pulse width.

*Juxtacellular labeling*

After recording, DRN neurons were subject to juxtacellular labeling. Briefly, its principle consists of applying stimulating on/off current pulses (1-10nA, 200ms) onto the membrane of the neuron which was entrained for 30-60s. One neuron was labeled per rat. After labeling, rats were maintained under general anaesthesia for approximately 1h, and then perfused intracardially with 0.9% saline solution, followed by 300 ml of 4% paraformaldehyde in 0.1 M phosphate buffer, pH 7.4. Hereafter, the brains were removed and cryo-protected in sucrose solution prior to sectioning.

*Computation of electrophysiological data*

Stimulation artifacts were removed from spike trains using standard spike-sorting techniques, including template-matching, principal component analysis, and supervised clustering (Spike2). Each trace was then visually inspected to ensure the quality of spike identification. Baseline firing rate was quantified during the final 60 s prior to stimulation. Interspike interval histograms were plotted for the baseline period, and regularity of firing was calculated for the same periods by standard coefficient of variation (COV; standard deviation of the interspike interval/mean interspike interval) analysis. Spike waveform width was calculated from the average of waveforms recorded during the final 60 s of baseline recording for each neuron, with width determined as the time between a 5% positive deviation from baseline to the return to baseline after a negative phase ^4, 5^.

Analysis of firing rate was based on previously established methods ^1^. In brief, firing rate was calculated for the last min prior to vmPFC stimulation as a control baseline, during the 2^nd^ and 5^th^ min of vmPFC HFS, and on the 5^th^ min following cessation of vmPFC stimulation. To categorize the response of individual neurons to vmPFC stimulation, firing rate histograms (10 s bin size) were plotted and compared to the mean ± 1 standard deviation (SD) of the firing rate of the last min of control baseline. The response of neurons to stimulation was classified as either inhibition or excitation or none. To be classified as an inhibitory effect, two consecutive bins during the 2^nd^ or 5^th^ min of stimulation were below the mean baseline firing rate −1 standard deviation. In contrast, if two consecutive bins were above the mean baseline firing rate +1 standard deviation of the mean, this was then classified as excitation.

**3. Histological processing**

*Immunohistochemistry*

One series of sections from the CMS study was taken for c-Fos immunohistochemistry using an anti-cFos antibody, rabbit polyclonal lgG (diluted 1:20 000; Santa Cruz Biotechnology Inc., Santa Cruz, Dallas, USA) as primary antibody for an overnight incubation. Meanwhile, another series was taken for 5-HT immunohistochemistry using an anti-5-HT rabbit antibody (1:50000, H. Steinbusch, Maastricht University, the Netherlands) for a three-night incubation. After rinsing, they were incubated with the secondary antibody (biotinylated donkey anti-rabbit biotin with dilution 1:400; Jackson Immunoresearch Laboratories Inc., Westgrove, USA) for 90 min. Subsequently, both c-Fos and 5-HT sections were incubated with an avidin-biotin-peroxidase complex (diluted 1:800 in Elite ABC-kit, Vectastatin; Vector, Burlingame, USA) for 2 h. To visualize the immune complex of horseradish peroxide reaction product, sections were incubated with 3,3’-diaminobenzidine tetrahydrochloride (DAB)/Nickel Chloride (NiCl_2_) solution. All sections were then mounted, dehydrated, and cover-slipped.

*Immunofluorescence staining*

After pre-blocking for 30 min in PBS-Triton (PBS-T) with 10% normal donkey serum, staining was carried out on 30 mm thick sections using rabbit anti-5-HT antibody (1:5000; H. Steinbusch, Maastricht University, the Netherlands) and goat anti-cFos (1:500; Santa Cruz Biotechnology Inc., Santa Cruz, Dallas, USA) as primary antibody in 5% normal donkey serum for 3 days incubation. After rinsing, sections were incubated in Alexa-fluor conjugated secondary antibodies (Alexa Fluor® 594 donkey anti-rabbit IgG and Alexa Fluor® 488 donkey anti-goat IgG; each 1:500; Vector Laboratories, Burlingame, CA, USA) for 2 h at room temperature. After sections were mounted on the Superfrost® micro-slides (VWM, Illinois, USA), blue-fluorescent DAPI was specifically used to counterstain the nuclei, and finally cover-slipped with Vectashield (Vector Laboratories, Burlingame, USA).

In the animals with juxtacellular labeling, brain sections containing the DRN regions were processed for immunofluorescence staining. After rinsing and pre-blocking for 30 min in PBS-T with 10% normal donkey serum, sections were incubated overnight in rabbit anti-5-HT antibody (1:6000; H. Steinbusch, Maastricht University, the Netherlands) in 2% normal donkey serum. After rinsing, sections were incubated for 2 h at room temperature in Alexa Fluor® 488 donkey anti-rabbit lgG (1:800, Invitrogen, Paisley, UK) and Alexa Fluor® 594 donkey anti-rabbit lgG conjugated streptavidin (1:1000, Invitrogen, Paisley, UK) in PBS-T containing 2% normal donkey serum. Sections were again rinsed and finally mounted on gelatin-coated slides and cover-slipped.

*Quantitative evaluation of c-Fos immunoreactive cells*

Systematic cell counts were performed on c-Fos immunoreactive (c-Fos-ir) cells in different brain regions of interest in the frontal cortex (Cg1, Cg2, VO, LO, PrL, IL), nucleus accumbens (NAc core, NAc shell), habenula (MHb, LHb), hippocampus (CA1, CA3, DG), hypothalamus (DMH, PeFLH, AH, PH, VMH, PaMP, PaLM), amygdala (MeA, CeA, LA, BLA), anterior and posterior part of periaqueductal gray (dmPAG, dlPAG, lPAG, vlPAG), deep cerebellar nuclei (Dent, IntMC, IntPC, Fast), vestibular nuclei (MVePC, MVeMC, SpVe), and dorsal raphe nuclei (DRN, DRV, DRVL). Our quantification approach was similar to a previously reported method, with minor modifications ^6, 7^. Photographs of the areas of interest were taken at 4X magnification and the boundaries of the areas of interest were delineated and measured. The counting of the numbers of c-Fos-ir cells was performed using the conventional image analysis program Image J (version 1.38, NIH, USA).

*Densitometry evaluation of 5-HT cells*

The mean Gray values of twenty 5-HT immunoreactive neurons in the DRN of each animal were assessed at 40X magnification using an imaging camera (Retiga Exi, Canada) connected to a bright-field microscope (Olympus BX61, Japan). The optical density (OD) was measured quantitatively using the conventional image analysis program Image J (version 1.38, NIH, USA) based on previously reported methods ^8^.

*Histological evaluation of juxtacellular labeled cells*

To analyze sections for presence of the 5-HT neurobiotin-labeled cells, photographs were taken using a digital camera (ORCA-ER (C4742-80), Hamamatsu, Japan) that connected to an epifluorescence microscope (Leica Microsystems, Milton Keynes, UK) and a laser-scanning confocal microscope (Carl Zeiss, Oberkochen, Germany).

**References:**

1. Hartung H, Tan SK, Steinbusch HM, Temel Y, Sharp T. High-frequency stimulation of the subthalamic nucleus inhibits the firing of juxtacellular labelled 5-HT-containing neurones. *Neuroscience*; **186:** 135-145.

2. Hajos M, Allers KA, Jennings K, Sharp T, Charette G, Sik A *et al.* Neurochemical identification of stereotypic burst-firing neurons in the rat dorsal raphe nucleus using juxtacellular labelling methods. *Eur J Neurosci* 2007; **25**(1)**:** 119-126.

3. Schweimer JV, Ungless MA. Phasic responses in dorsal raphe serotonin neurons to noxious stimuli. *Neuroscience*; **171**(4)**:** 1209-1215.

4. Allers KA, Sharp T. Neurochemical and anatomical identification of fast- and slow-firing neurones in the rat dorsal raphe nucleus using juxtacellular labelling methods in vivo. *Neuroscience* 2003; **122**(1)**:** 193-204.

5. Varga V, Kocsis B, Sharp T. Electrophysiological evidence for convergence of inputs from the medial prefrontal cortex and lateral habenula on single neurons in the dorsal raphe nucleus. *Eur J Neurosci* 2003; **17**(2)**:** 280-286.

6. Lim LW, Temel Y, Sesia T, Vlamings R, Visser-Vandewalle V, Steinbusch HW *et al.* Buspirone induced acute and chronic changes of neural activation in the periaqueductal gray of rats. *Neuroscience* 2008; **155**(1)**:** 164-173.

7. Hestermann D, Temel Y, Blokland A, Lim LW. Acute serotonergic treatment changes the relation between anxiety and HPA-axis functioning and periaqueductal gray activation. *Behav Brain Res* 2014; **273:** 155-165.

8. Jahanshahi A, Lim LW, Steinbusch HW, Visser-Vandewalle V, Temel Y. Buspirone-induced changes in the serotonergic and non-serotonergic cells in the dorsal raphe nucleus of rats. *Neurosci Lett*; **473**(2)**:** 136-140.
