# Supplementary material for: Electrical stimulation alleviates depressive-like behaviors of rats: investigation of brain targets and potential mechanisms
Source: Transl Psychiatry. 2015 Mar 31;5(3):e535–. doi: 10.1038/tp.2015.24 (PMC4354354; doi:10.1038/tp.2015.24)
Supplement: Supplementary Tables [file tp201524x2.ppt]

## Slide 1
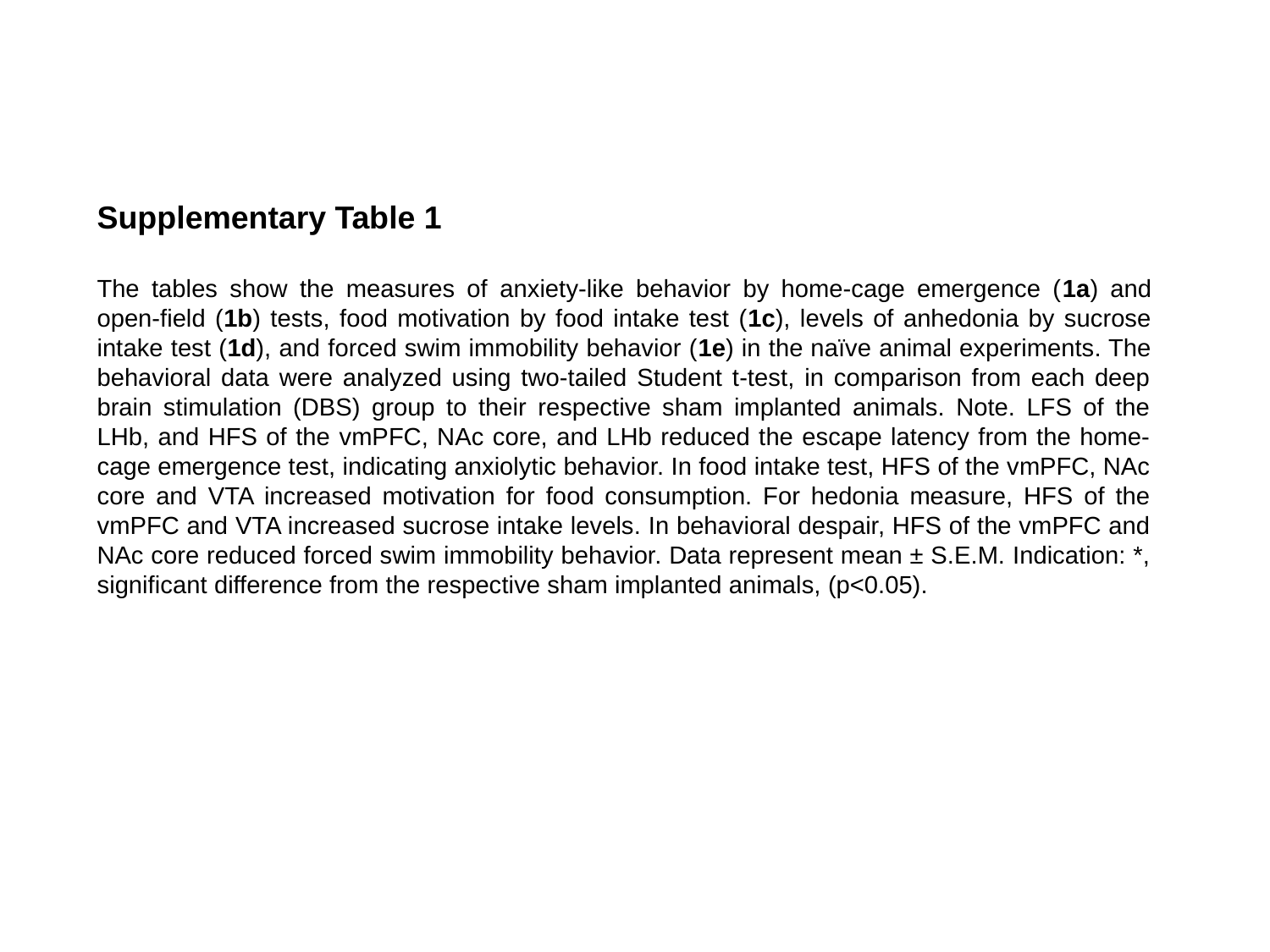

Supplementary Table 1
The tables show the measures of anxiety-like behavior by home-cage emergence (1a) and open-field (1b) tests, food motivation by food intake test (1c), levels of anhedonia by sucrose intake test (1d), and forced swim immobility behavior (1e) in the naïve animal experiments. The behavioral data were analyzed using two-tailed Student t-test, in comparison from each deep brain stimulation (DBS) group to their respective sham implanted animals. Note. LFS of the LHb, and HFS of the vmPFC, NAc core, and LHb reduced the escape latency from the home-cage emergence test, indicating anxiolytic behavior. In food intake test, HFS of the vmPFC, NAc core and VTA increased motivation for food consumption. For hedonia measure, HFS of the vmPFC and VTA increased sucrose intake levels. In behavioral despair, HFS of the vmPFC and NAc core reduced forced swim immobility behavior. Data represent mean ± S.E.M. Indication: *, significant difference from the respective sham implanted animals, (p<0.05).

## Slide 2
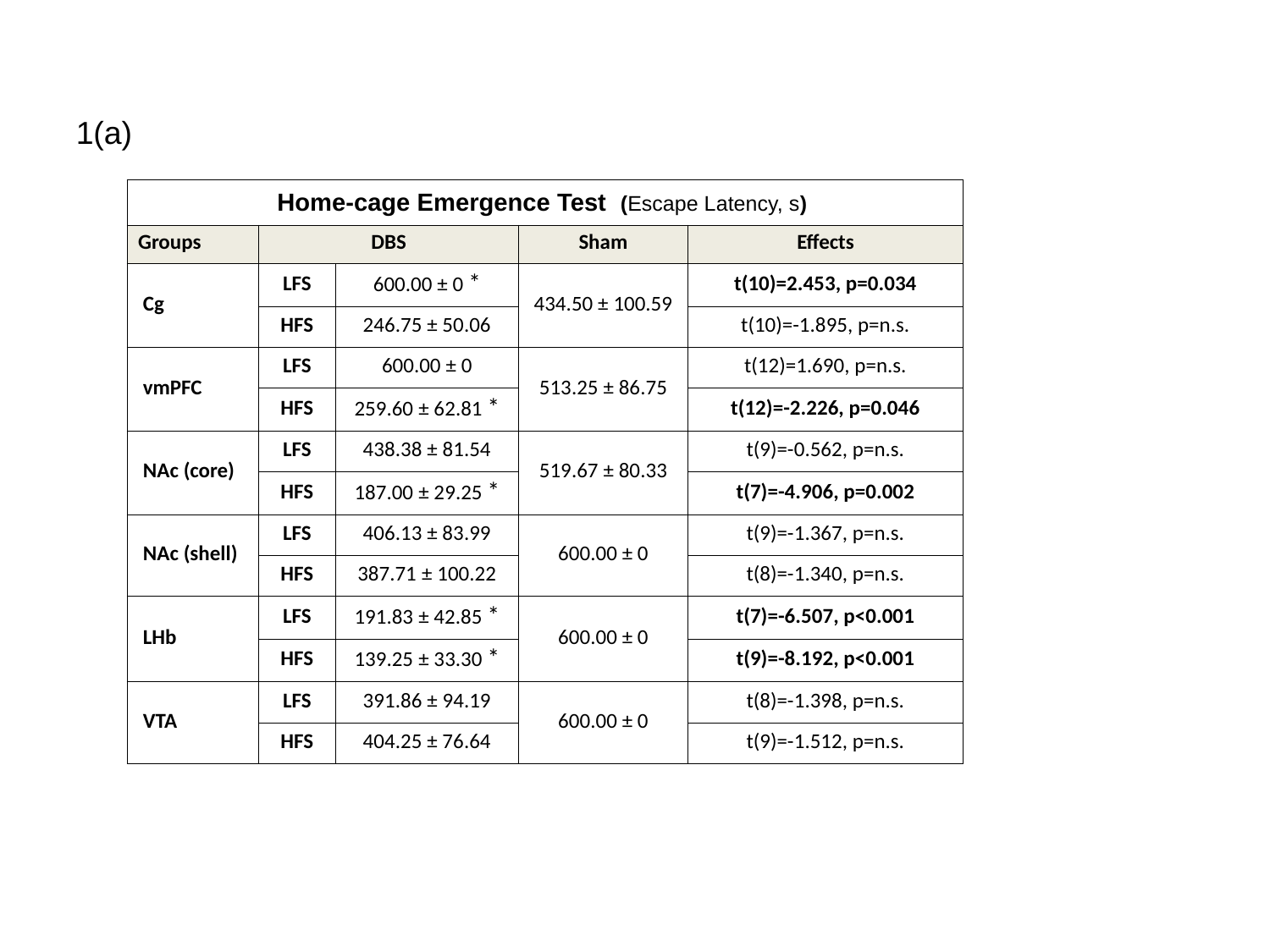

1(a)
| Home-cage Emergence Test (Escape Latency, s) | | | | |
| --- | --- | --- | --- | --- |
| Groups | DBS | | Sham | Effects |
| Cg | LFS | 600.00 ± 0 \* | 434.50 ± 100.59 | t(10)=2.453, p=0.034 |
| | HFS | 246.75 ± 50.06 | | t(10)=-1.895, p=n.s. |
| vmPFC | LFS | 600.00 ± 0 | 513.25 ± 86.75 | t(12)=1.690, p=n.s. |
| | HFS | 259.60 ± 62.81 \* | | t(12)=-2.226, p=0.046 |
| NAc (core) | LFS | 438.38 ± 81.54 | 519.67 ± 80.33 | t(9)=-0.562, p=n.s. |
| | HFS | 187.00 ± 29.25 \* | | t(7)=-4.906, p=0.002 |
| NAc (shell) | LFS | 406.13 ± 83.99 | 600.00 ± 0 | t(9)=-1.367, p=n.s. |
| | HFS | 387.71 ± 100.22 | | t(8)=-1.340, p=n.s. |
| LHb | LFS | 191.83 ± 42.85 \* | 600.00 ± 0 | t(7)=-6.507, p<0.001 |
| | HFS | 139.25 ± 33.30 \* | | t(9)=-8.192, p<0.001 |
| VTA | LFS | 391.86 ± 94.19 | 600.00 ± 0 | t(8)=-1.398, p=n.s. |
| | HFS | 404.25 ± 76.64 | | t(9)=-1.512, p=n.s. |

## Slide 3
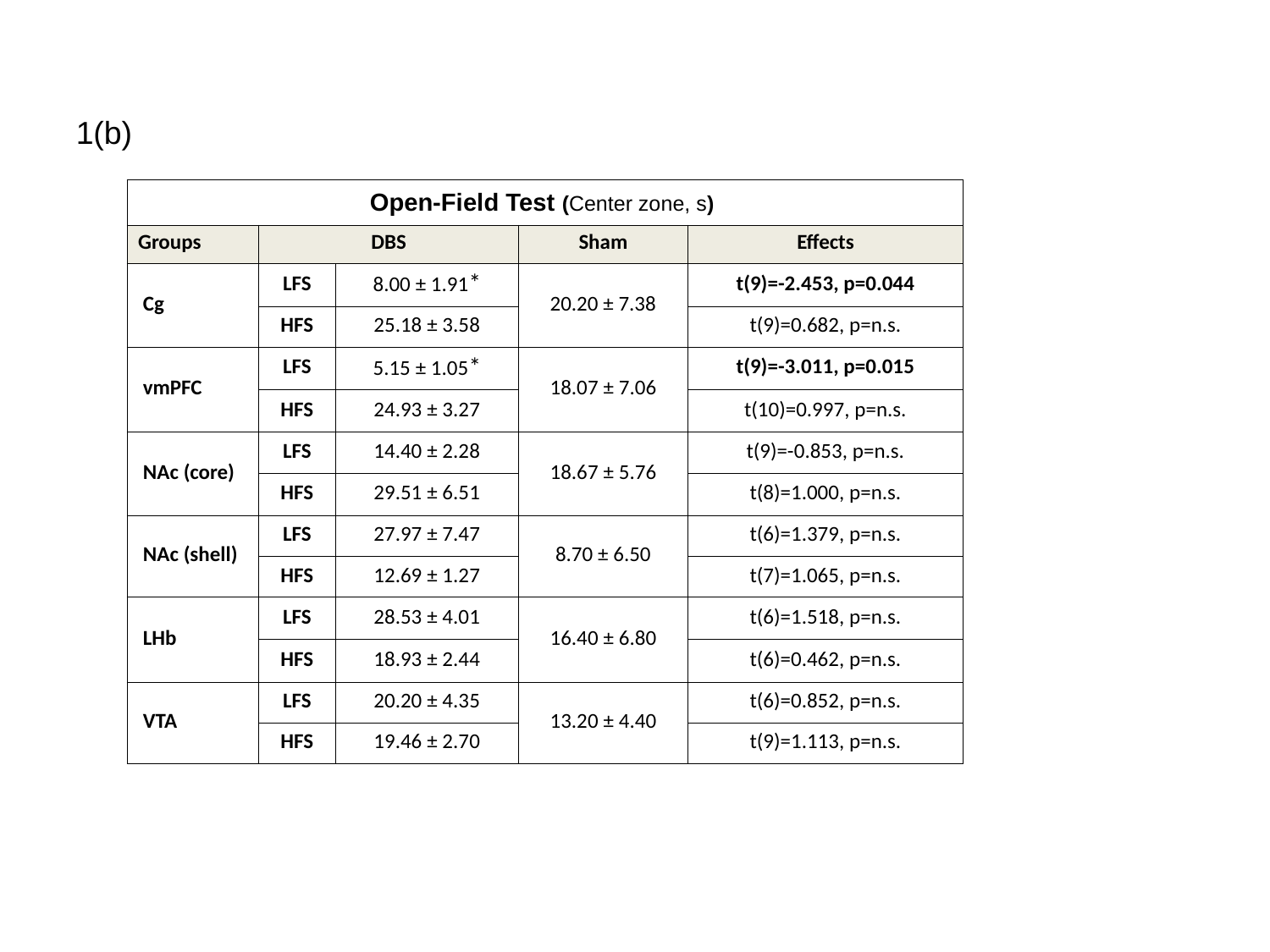

1(b)
| Open-Field Test (Center zone, s) | | | | |
| --- | --- | --- | --- | --- |
| Groups | DBS | | Sham | Effects |
| Cg | LFS | 8.00 ± 1.91\* | 20.20 ± 7.38 | t(9)=-2.453, p=0.044 |
| | HFS | 25.18 ± 3.58 | | t(9)=0.682, p=n.s. |
| vmPFC | LFS | 5.15 ± 1.05\* | 18.07 ± 7.06 | t(9)=-3.011, p=0.015 |
| | HFS | 24.93 ± 3.27 | | t(10)=0.997, p=n.s. |
| NAc (core) | LFS | 14.40 ± 2.28 | 18.67 ± 5.76 | t(9)=-0.853, p=n.s. |
| | HFS | 29.51 ± 6.51 | | t(8)=1.000, p=n.s. |
| NAc (shell) | LFS | 27.97 ± 7.47 | 8.70 ± 6.50 | t(6)=1.379, p=n.s. |
| | HFS | 12.69 ± 1.27 | | t(7)=1.065, p=n.s. |
| LHb | LFS | 28.53 ± 4.01 | 16.40 ± 6.80 | t(6)=1.518, p=n.s. |
| | HFS | 18.93 ± 2.44 | | t(6)=0.462, p=n.s. |
| VTA | LFS | 20.20 ± 4.35 | 13.20 ± 4.40 | t(6)=0.852, p=n.s. |
| | HFS | 19.46 ± 2.70 | | t(9)=1.113, p=n.s. |

## Slide 4
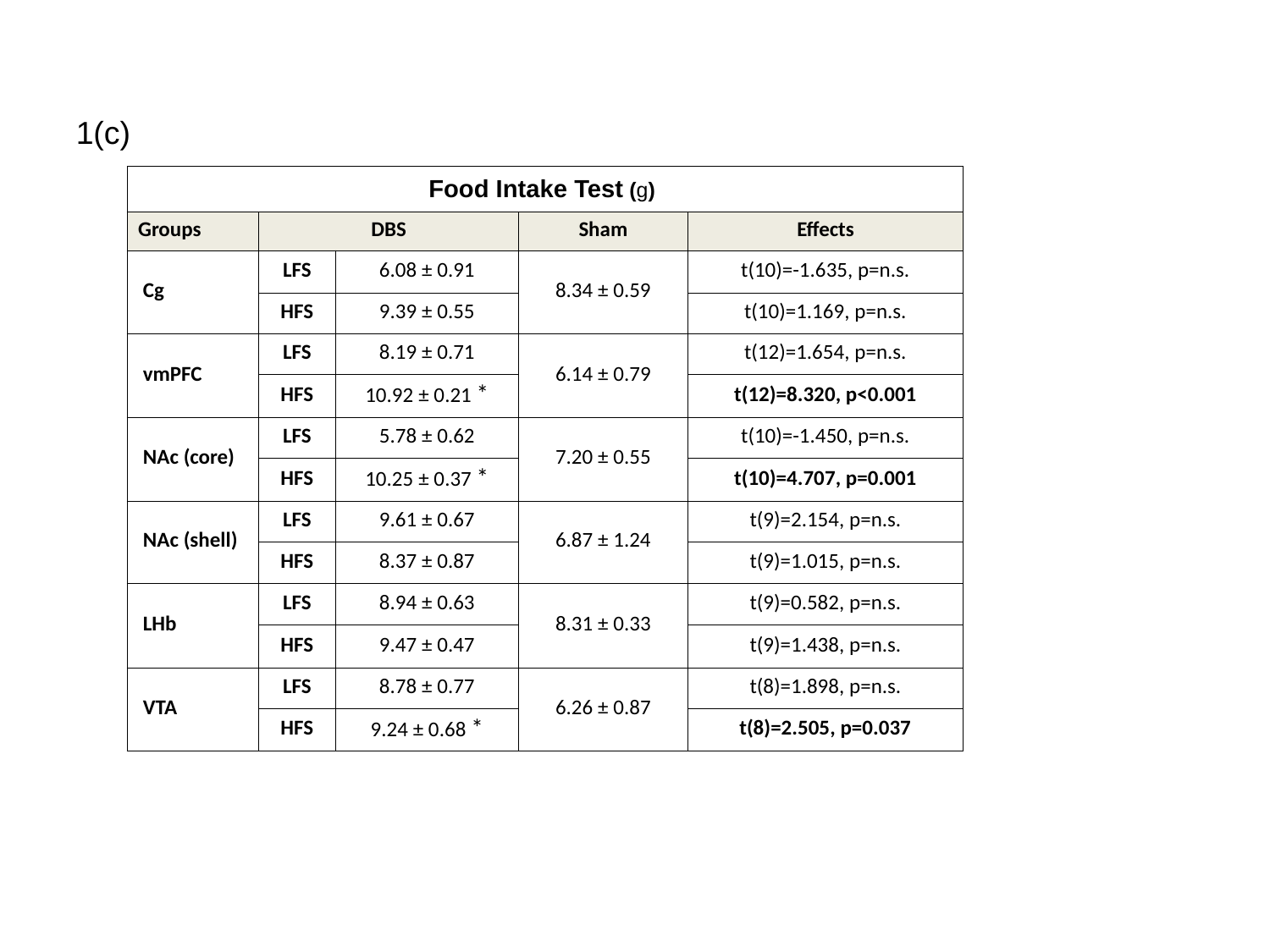

1(c)
| Food Intake Test (g) | | | | |
| --- | --- | --- | --- | --- |
| Groups | DBS | | Sham | Effects |
| Cg | LFS | 6.08 ± 0.91 | 8.34 ± 0.59 | t(10)=-1.635, p=n.s. |
| | HFS | 9.39 ± 0.55 | | t(10)=1.169, p=n.s. |
| vmPFC | LFS | 8.19 ± 0.71 | 6.14 ± 0.79 | t(12)=1.654, p=n.s. |
| | HFS | 10.92 ± 0.21 \* | | t(12)=8.320, p<0.001 |
| NAc (core) | LFS | 5.78 ± 0.62 | 7.20 ± 0.55 | t(10)=-1.450, p=n.s. |
| | HFS | 10.25 ± 0.37 \* | | t(10)=4.707, p=0.001 |
| NAc (shell) | LFS | 9.61 ± 0.67 | 6.87 ± 1.24 | t(9)=2.154, p=n.s. |
| | HFS | 8.37 ± 0.87 | | t(9)=1.015, p=n.s. |
| LHb | LFS | 8.94 ± 0.63 | 8.31 ± 0.33 | t(9)=0.582, p=n.s. |
| | HFS | 9.47 ± 0.47 | | t(9)=1.438, p=n.s. |
| VTA | LFS | 8.78 ± 0.77 | 6.26 ± 0.87 | t(8)=1.898, p=n.s. |
| | HFS | 9.24 ± 0.68 \* | | t(8)=2.505, p=0.037 |

## Slide 5
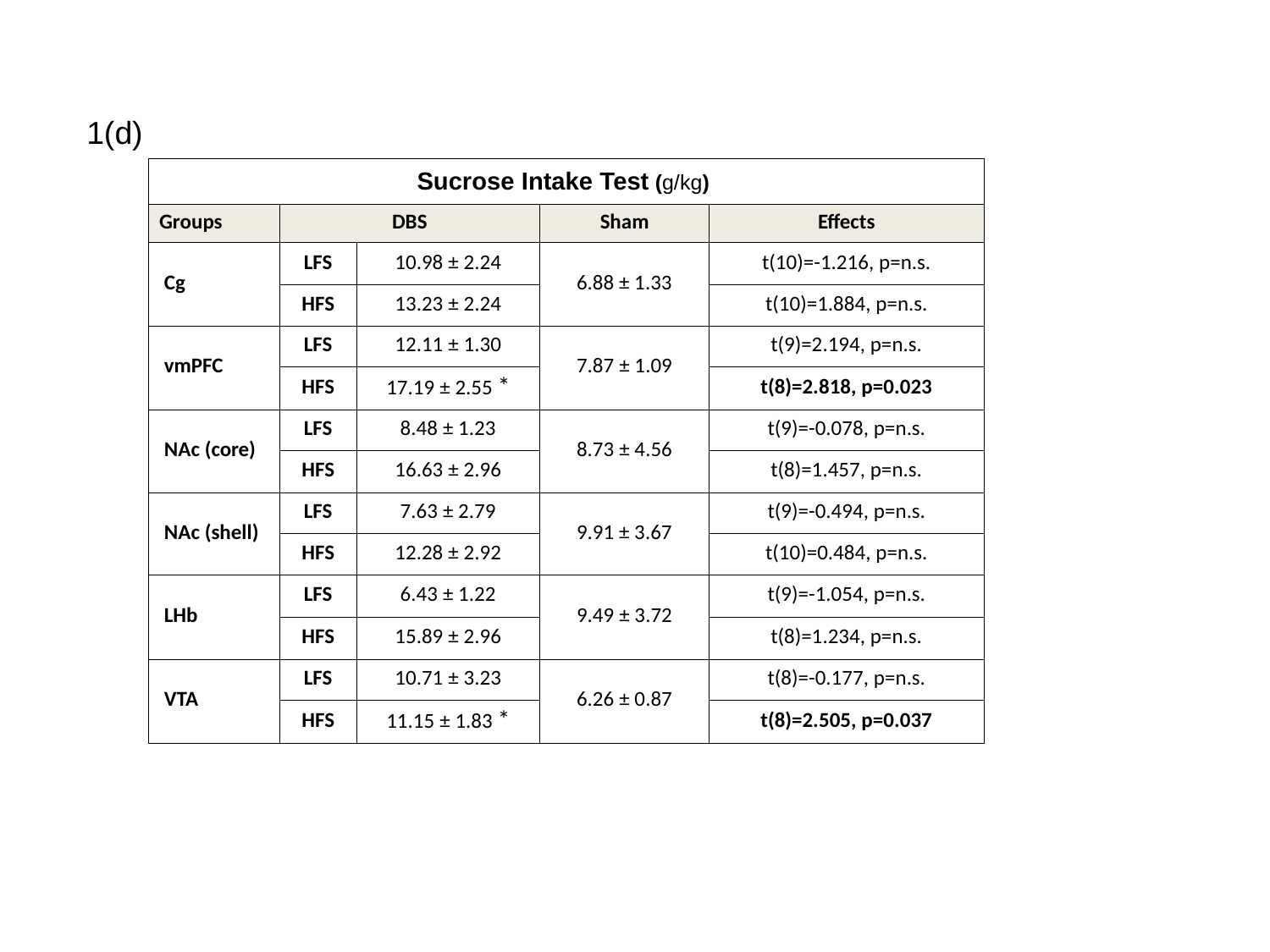

1(d)
| Sucrose Intake Test (g/kg) | | | | |
| --- | --- | --- | --- | --- |
| Groups | DBS | | Sham | Effects |
| Cg | LFS | 10.98 ± 2.24 | 6.88 ± 1.33 | t(10)=-1.216, p=n.s. |
| | HFS | 13.23 ± 2.24 | | t(10)=1.884, p=n.s. |
| vmPFC | LFS | 12.11 ± 1.30 | 7.87 ± 1.09 | t(9)=2.194, p=n.s. |
| | HFS | 17.19 ± 2.55 \* | | t(8)=2.818, p=0.023 |
| NAc (core) | LFS | 8.48 ± 1.23 | 8.73 ± 4.56 | t(9)=-0.078, p=n.s. |
| | HFS | 16.63 ± 2.96 | | t(8)=1.457, p=n.s. |
| NAc (shell) | LFS | 7.63 ± 2.79 | 9.91 ± 3.67 | t(9)=-0.494, p=n.s. |
| | HFS | 12.28 ± 2.92 | | t(10)=0.484, p=n.s. |
| LHb | LFS | 6.43 ± 1.22 | 9.49 ± 3.72 | t(9)=-1.054, p=n.s. |
| | HFS | 15.89 ± 2.96 | | t(8)=1.234, p=n.s. |
| VTA | LFS | 10.71 ± 3.23 | 6.26 ± 0.87 | t(8)=-0.177, p=n.s. |
| | HFS | 11.15 ± 1.83 \* | | t(8)=2.505, p=0.037 |

## Slide 6
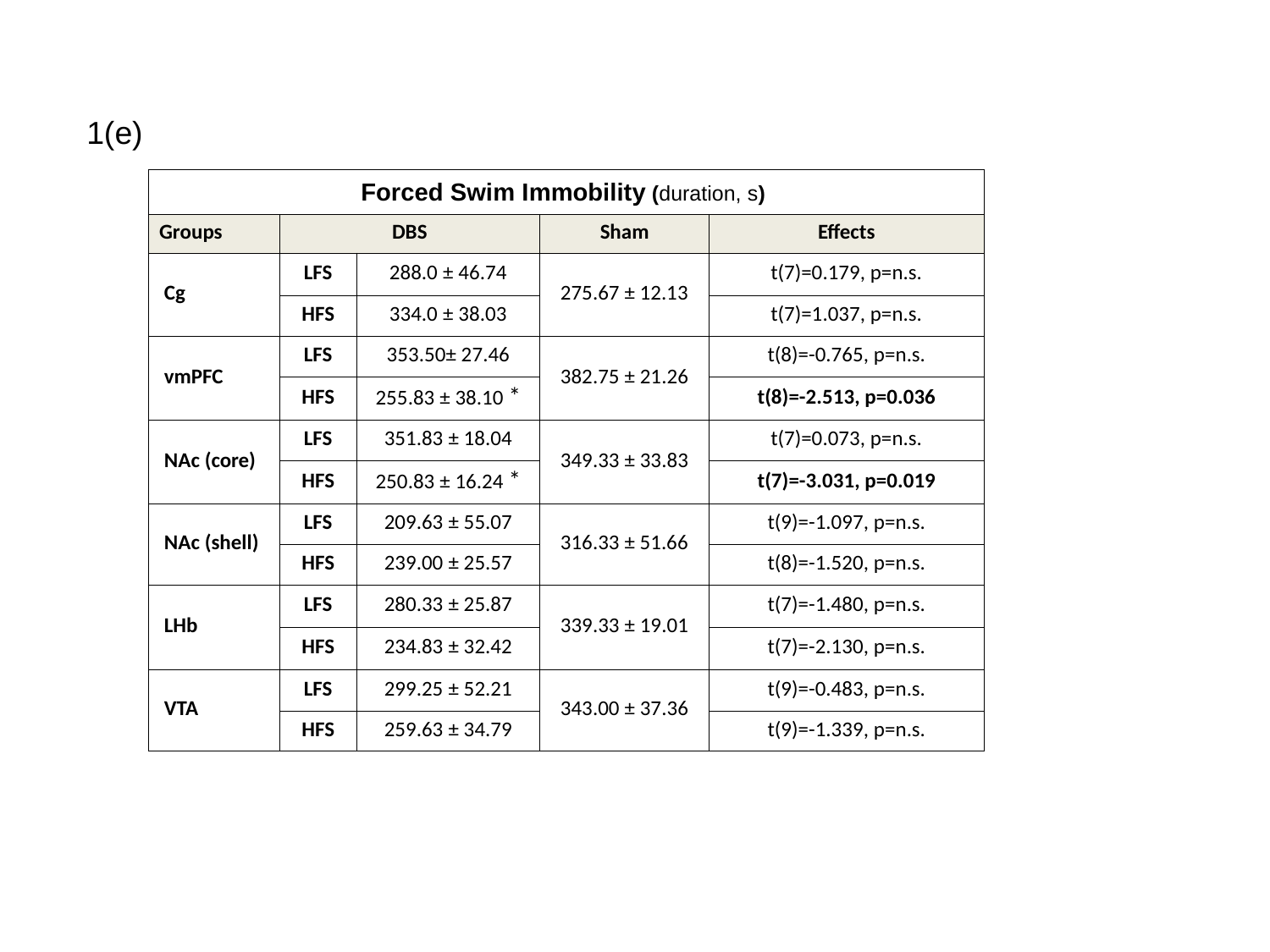

1(e)
| Forced Swim Immobility (duration, s) | | | | |
| --- | --- | --- | --- | --- |
| Groups | DBS | | Sham | Effects |
| Cg | LFS | 288.0 ± 46.74 | 275.67 ± 12.13 | t(7)=0.179, p=n.s. |
| | HFS | 334.0 ± 38.03 | | t(7)=1.037, p=n.s. |
| vmPFC | LFS | 353.50± 27.46 | 382.75 ± 21.26 | t(8)=-0.765, p=n.s. |
| | HFS | 255.83 ± 38.10 \* | | t(8)=-2.513, p=0.036 |
| NAc (core) | LFS | 351.83 ± 18.04 | 349.33 ± 33.83 | t(7)=0.073, p=n.s. |
| | HFS | 250.83 ± 16.24 \* | | t(7)=-3.031, p=0.019 |
| NAc (shell) | LFS | 209.63 ± 55.07 | 316.33 ± 51.66 | t(9)=-1.097, p=n.s. |
| | HFS | 239.00 ± 25.57 | | t(8)=-1.520, p=n.s. |
| LHb | LFS | 280.33 ± 25.87 | 339.33 ± 19.01 | t(7)=-1.480, p=n.s. |
| | HFS | 234.83 ± 32.42 | | t(7)=-2.130, p=n.s. |
| VTA | LFS | 299.25 ± 52.21 | 343.00 ± 37.36 | t(9)=-0.483, p=n.s. |
| | HFS | 259.63 ± 34.79 | | t(9)=-1.339, p=n.s. |

## Slide 7
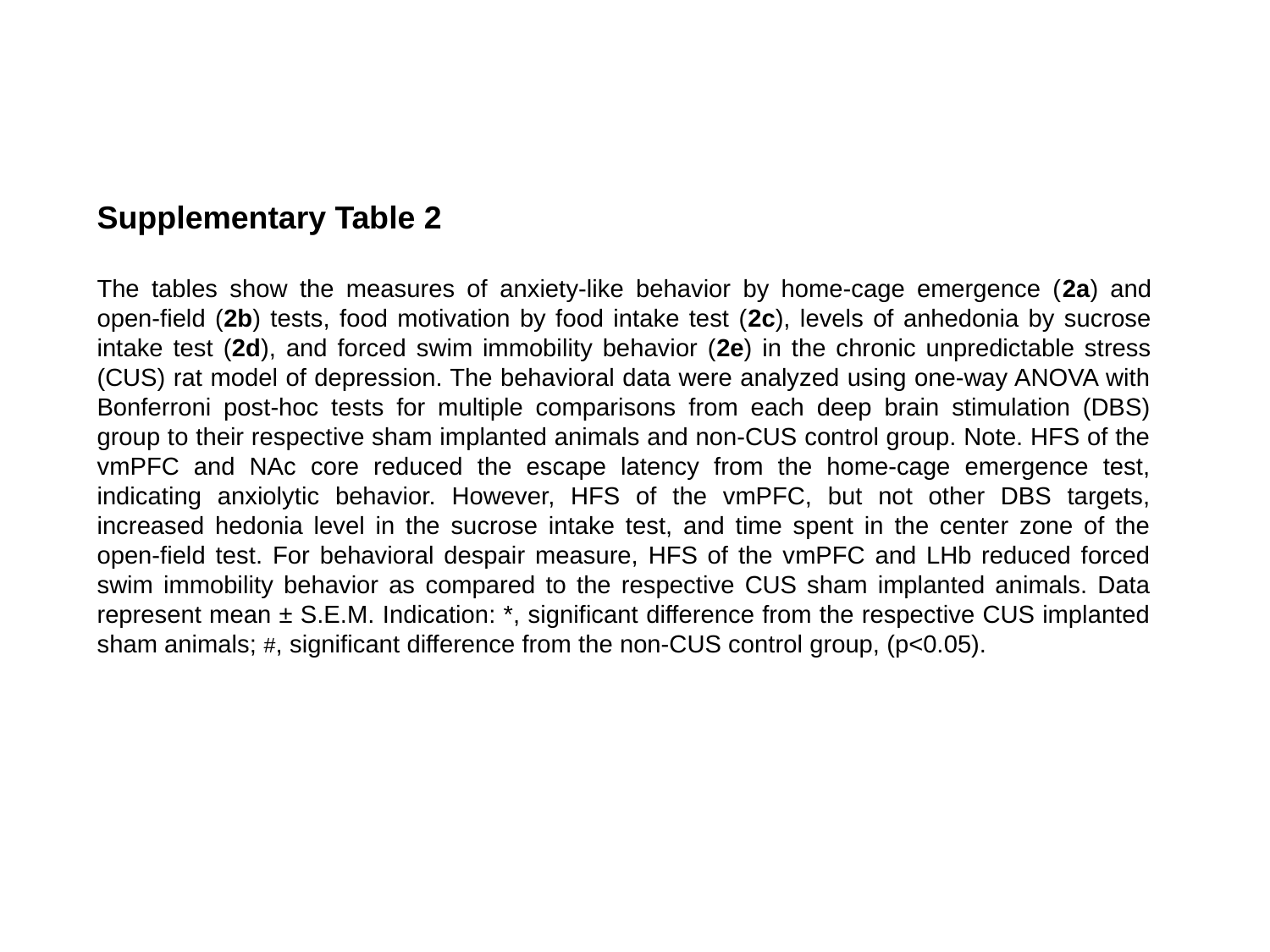

Supplementary Table 2
The tables show the measures of anxiety-like behavior by home-cage emergence (2a) and open-field (2b) tests, food motivation by food intake test (2c), levels of anhedonia by sucrose intake test (2d), and forced swim immobility behavior (2e) in the chronic unpredictable stress (CUS) rat model of depression. The behavioral data were analyzed using one-way ANOVA with Bonferroni post-hoc tests for multiple comparisons from each deep brain stimulation (DBS) group to their respective sham implanted animals and non-CUS control group. Note. HFS of the vmPFC and NAc core reduced the escape latency from the home-cage emergence test, indicating anxiolytic behavior. However, HFS of the vmPFC, but not other DBS targets, increased hedonia level in the sucrose intake test, and time spent in the center zone of the open-field test. For behavioral despair measure, HFS of the vmPFC and LHb reduced forced swim immobility behavior as compared to the respective CUS sham implanted animals. Data represent mean ± S.E.M. Indication: *, significant difference from the respective CUS implanted sham animals; #, significant difference from the non-CUS control group, (p<0.05).

## Slide 8
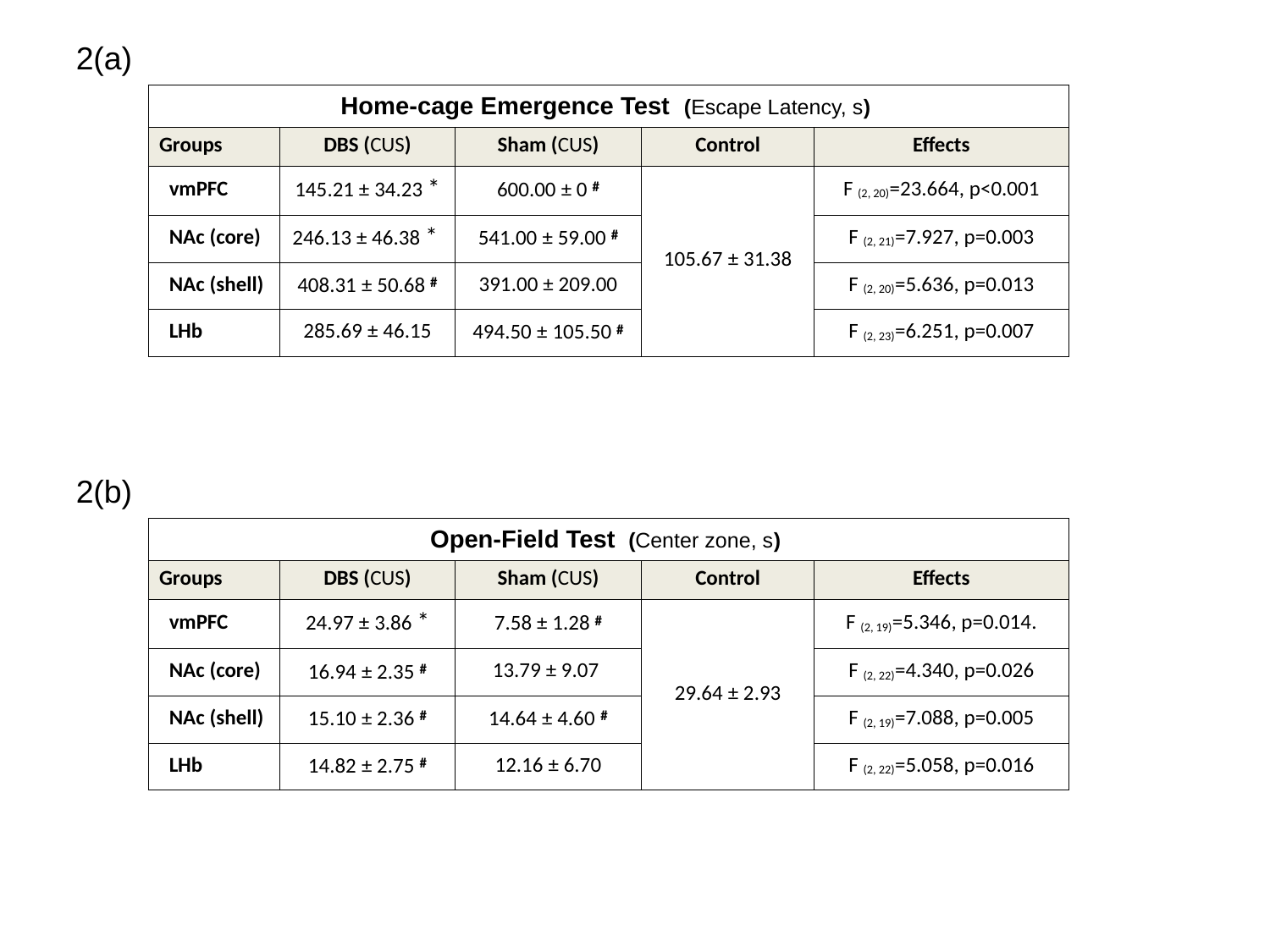

2(a)
| Home-cage Emergence Test (Escape Latency, s) | | | | |
| --- | --- | --- | --- | --- |
| Groups | DBS (CUS) | Sham (CUS) | Control | Effects |
| vmPFC | 145.21 ± 34.23 \* | 600.00 ± 0 # | 105.67 ± 31.38 | F (2, 20)=23.664, p<0.001 |
| NAc (core) | 246.13 ± 46.38 \* | 541.00 ± 59.00 # | | F (2, 21)=7.927, p=0.003 |
| NAc (shell) | 408.31 ± 50.68 # | 391.00 ± 209.00 | | F (2, 20)=5.636, p=0.013 |
| LHb | 285.69 ± 46.15 | 494.50 ± 105.50 # | | F (2, 23)=6.251, p=0.007 |
2(b)
| Open-Field Test (Center zone, s) | | | | |
| --- | --- | --- | --- | --- |
| Groups | DBS (CUS) | Sham (CUS) | Control | Effects |
| vmPFC | 24.97 ± 3.86 \* | 7.58 ± 1.28 # | 29.64 ± 2.93 | F (2, 19)=5.346, p=0.014. |
| NAc (core) | 16.94 ± 2.35 # | 13.79 ± 9.07 | | F (2, 22)=4.340, p=0.026 |
| NAc (shell) | 15.10 ± 2.36 # | 14.64 ± 4.60 # | | F (2, 19)=7.088, p=0.005 |
| LHb | 14.82 ± 2.75 # | 12.16 ± 6.70 | | F (2, 22)=5.058, p=0.016 |

## Slide 9
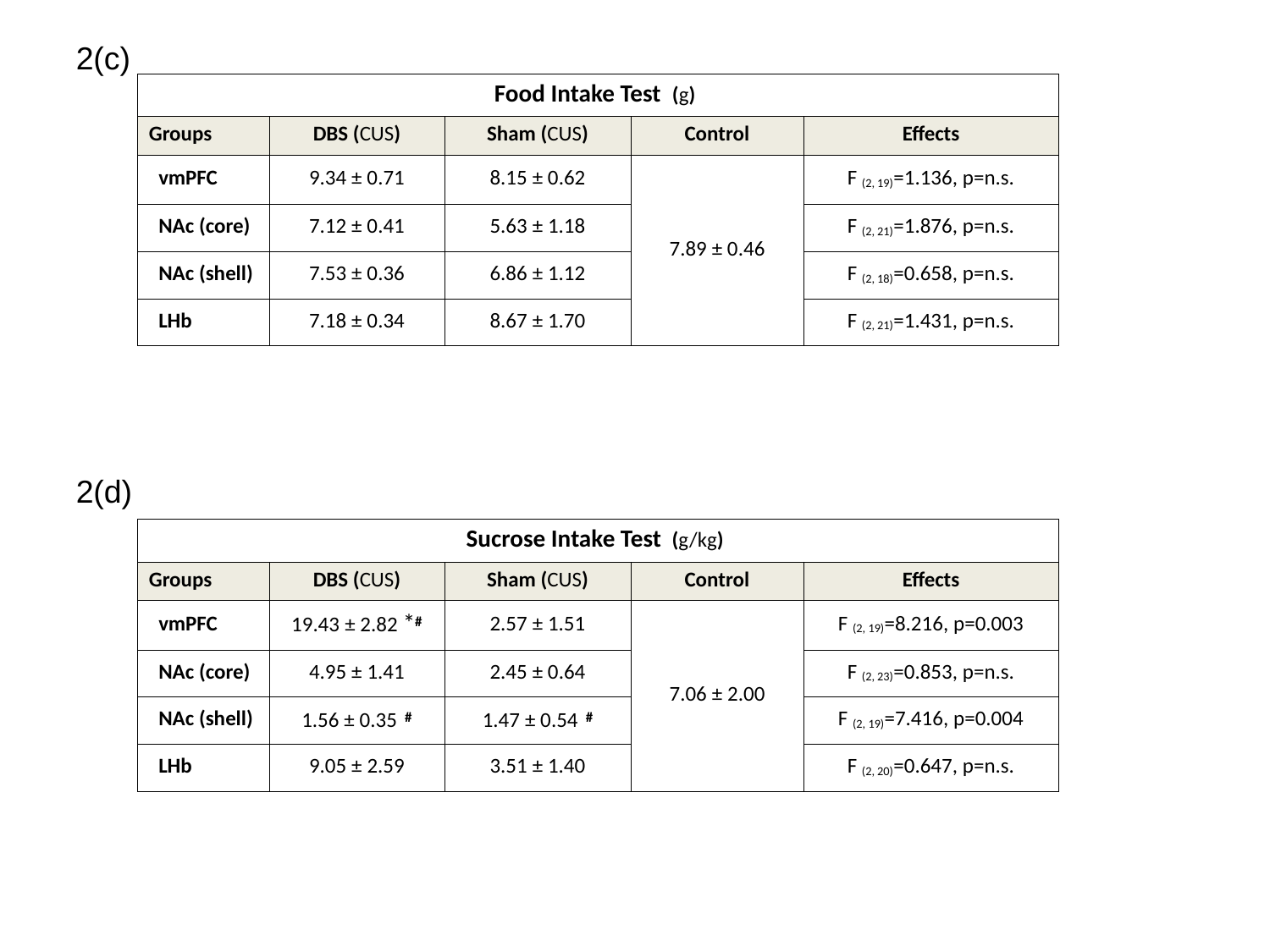

2(c)
| Food Intake Test (g) | | | | |
| --- | --- | --- | --- | --- |
| Groups | DBS (CUS) | Sham (CUS) | Control | Effects |
| vmPFC | 9.34 ± 0.71 | 8.15 ± 0.62 | 7.89 ± 0.46 | F (2, 19)=1.136, p=n.s. |
| NAc (core) | 7.12 ± 0.41 | 5.63 ± 1.18 | | F (2, 21)=1.876, p=n.s. |
| NAc (shell) | 7.53 ± 0.36 | 6.86 ± 1.12 | | F (2, 18)=0.658, p=n.s. |
| LHb | 7.18 ± 0.34 | 8.67 ± 1.70 | | F (2, 21)=1.431, p=n.s. |
2(d)
| Sucrose Intake Test (g/kg) | | | | |
| --- | --- | --- | --- | --- |
| Groups | DBS (CUS) | Sham (CUS) | Control | Effects |
| vmPFC | 19.43 ± 2.82 \*# | 2.57 ± 1.51 | 7.06 ± 2.00 | F (2, 19)=8.216, p=0.003 |
| NAc (core) | 4.95 ± 1.41 | 2.45 ± 0.64 | | F (2, 23)=0.853, p=n.s. |
| NAc (shell) | 1.56 ± 0.35 # | 1.47 ± 0.54 # | | F (2, 19)=7.416, p=0.004 |
| LHb | 9.05 ± 2.59 | 3.51 ± 1.40 | | F (2, 20)=0.647, p=n.s. |

## Slide 10
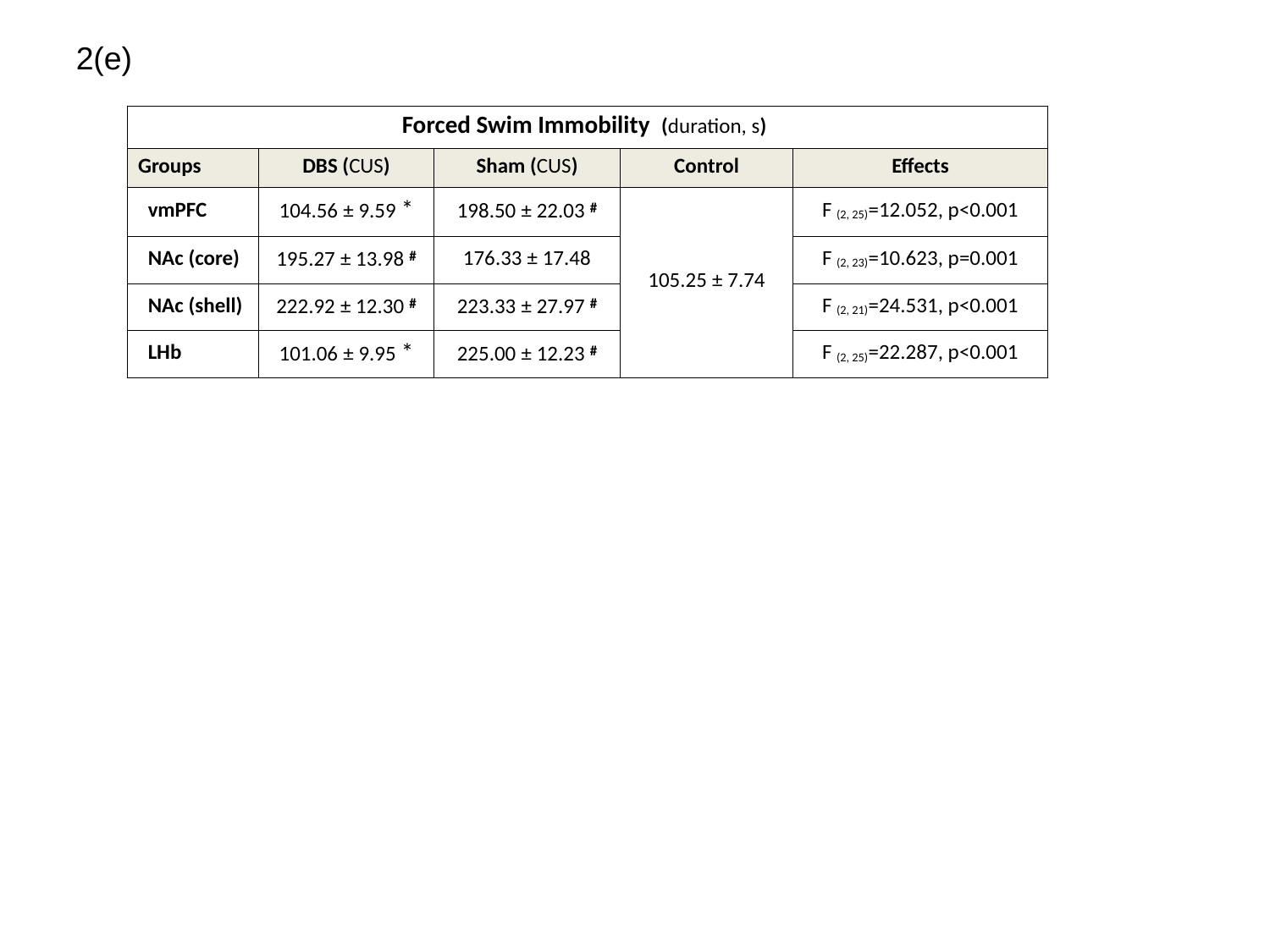

2(e)
| Forced Swim Immobility (duration, s) | | | | |
| --- | --- | --- | --- | --- |
| Groups | DBS (CUS) | Sham (CUS) | Control | Effects |
| vmPFC | 104.56 ± 9.59 \* | 198.50 ± 22.03 # | 105.25 ± 7.74 | F (2, 25)=12.052, p<0.001 |
| NAc (core) | 195.27 ± 13.98 # | 176.33 ± 17.48 | | F (2, 23)=10.623, p=0.001 |
| NAc (shell) | 222.92 ± 12.30 # | 223.33 ± 27.97 # | | F (2, 21)=24.531, p<0.001 |
| LHb | 101.06 ± 9.95 \* | 225.00 ± 12.23 # | | F (2, 25)=22.287, p<0.001 |
